# A hybrid long short-term memory with generalized additive model and post-hoc explainable artificial intelligence with causal inference for air pollutants prediction in Kimberley, South Africa

**DOI:** 10.3389/frai.2025.1620019

**Published:** 2025-08-04

**Authors:** Israel Edem Agbehadji, Ibidun Christiana Obagbuwa

**Affiliations:** ^1^Centre for Global Change, Sol Plaatje University, Kimberley, South Africa; ^2^Department of Computer Science and Information Technology, Faculty of Natural and Applied Sciences, Sol Plaatje University, Kimberley, South Africa

**Keywords:** generative additive model, post-hoc explanation, local interpretable model-agnostic explanation, deep learning, causal inference

## Abstract

The study addresses the problem of nonlinear characteristics of common air pollutants by proposing a deep learning time-series model based on the long short-term memory (LSTM) integrated with a generalized additive model (GAM). LSTM model captures both nonlinear relationships and temporal long-term dependencies in time-series data, and GAM provides insight into the statistical relationship between selected features and the target pollutant. The post-hoc eXplainable artificial intelligence (xAI) technique, local interpretable model-agnostic explanation (LIME), further explains the nonlinearity. Finally, causal inference was determined on the impact of the air pollutants relationship, thereby offering further interpretability in which deep learning models are deficient. Meteorological and air pollutant statistical records were leveraged from a Hantam (Karoo) air monitoring station in South Africa, and through a random sampling approach, synthetic data were generated for the city of Kimberley. The model was evaluated with the mean squared error (MSE), root mean squared error (RMSE) and mean absolute error (MAE) for different time-steps. The proposed referred to as long short-term memory generalized additive model based post-hoc eXplainable Artificial Intelligence (LSTM-GAM_xAI) model with a 10-day time-step and 5-day time-step for multiple pollutants prediction guaranteed least MSE. Though the causal effect analysis show no *p*-values (>0.88) for variables, the experiment results show that LSTM-GAM-xAI guaranteed the lowest MSE values across different time-steps.

## 1 Introduction

The prediction of air pollutant concentration continues to receive attention from the research community due to the health risk impact of high levels of air pollutant concentrations. Despite the World Health Organization (WHO) standard limits on the allowable air pollutant concentration in the environment, the problem of air pollution persists in some countries. Some of the sources of these air pollutants include power plants and cement factories, and the transport sector, which are known to emit Nitrogen Dioxide (NO_2_) into the atmosphere (Tu et al., 2023).

Air is a combination of gaseous components, and understanding the correlation is key, which can equally be achieved with machine learning or deep learning time-series models. Deep learning models for time-series analysis find patterns in the air pollutant dataset to help understand the nature of correlation through their network architecture. For instance, deep learning models such as the long short-term memory model (LSTM) use their gated mechanism to filter the necessary data inputs to produce refined output. Generally, LSTM is one of the commonly used models for time-series analysis, which requires training and testing with volumes of data. Other variants of deep learning models includes bi-directional LSTM (BiLSTM), gated recurrent unit (GRU), bi-gated recurrent unit (BiGRU), and one-dimensional convolutional neural network (1DCNN). These model, among other time-series models, captures complex nonlinear and temporal long-term dependencies in air pollutants (Agbehadji and Obagbuwa, 2025a). Mostly, air pollutants exhibit dynamic tendencies that make their modeling challenging. In this regard, the modeling of air quality tasks is not immune to errors that could impact on model’s performance. Several attempts have been made by researchers to address the errors from either a data or a model structure perspective. For instance, the use of an adaptive Kalman filter in an LSTM model to address prediction performance and also noise in the dataset (Agbehadji and Obagbuwa, 2025b). Again, the ensemble Kalman Filter was integrated with machine learning and deep learning models for long-term forecasting to reduce the level of uncertainty in data-driven models (Cheng et al., 2023). Therefore, hybrid models have contributed greatly to enhancing predictive accuracy in most prediction tasks. Wahiduzzaman and Yeasmin (2024) enhanced the predictive capability of deep learning models by utilizing a generalized additive model (GAM) to smooth the transformation of predictors. GAM is a machine learning model that helps to capture nonlinearity in data through its smoothing function capability (Tyralis and Papacharalampous, 2024). One of the potential of GAM is that it help understand problems within relevant domains such as air pollution to help stakeholders formulate their policies. Again, it help to bridge the gap between traditional statistical models and machine learning or deep learning models which make it a very valuable tool in xAI. While the role of neural-based GAM has been identified to provide interpretable and transparent deep learning models (Ortega-Fernandez et al., 2024), understanding the causal effect of air pollutants in a predictive task remains a challenge.

**Figure 1:**
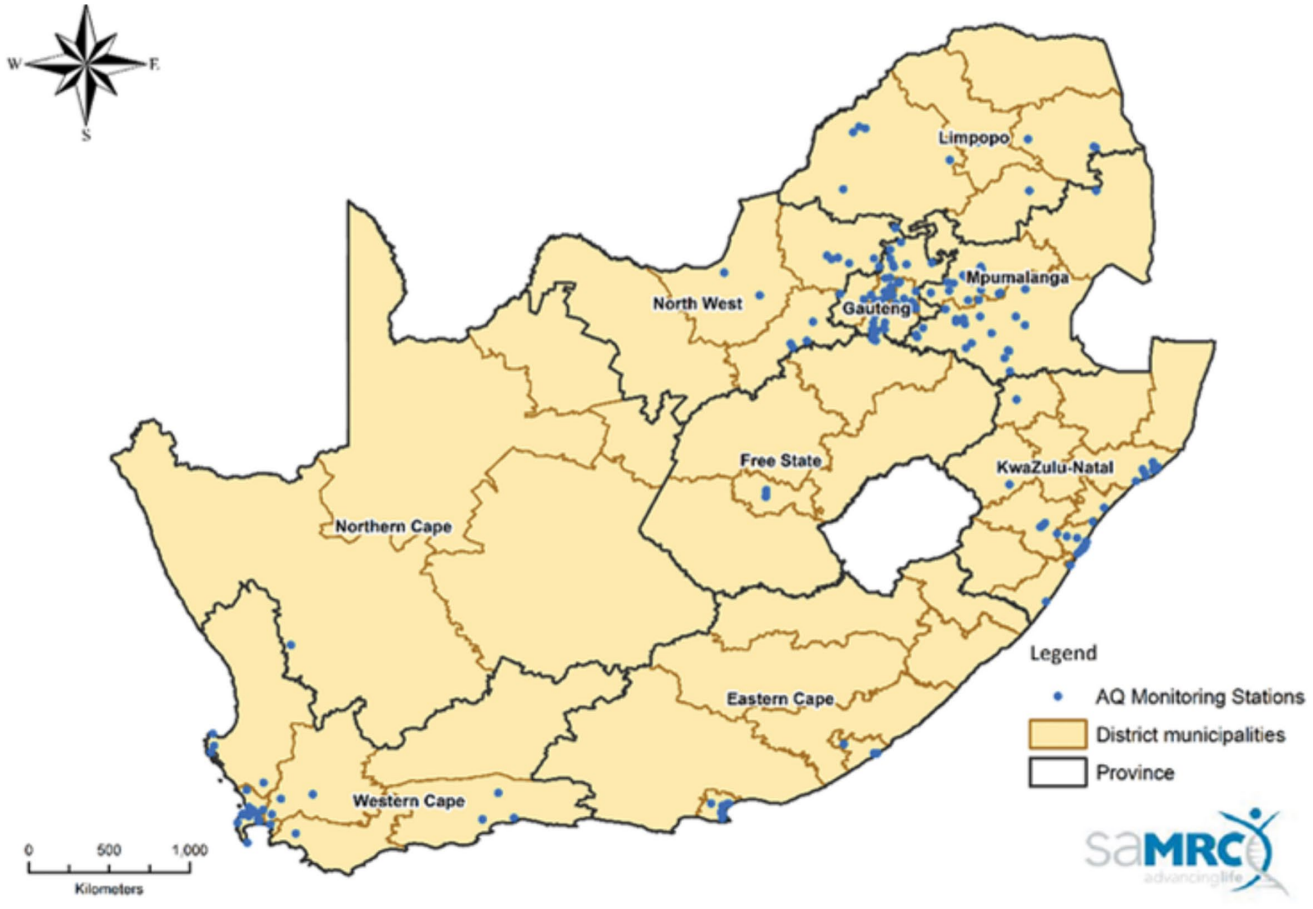
Map of South Africa showing Air quality monitoring stations in South Africa as of January 2023.

To solve the aforementioned challenge, this study attempts to develop a novel air predictive model for Kimberley. Kimberley is located in the Northern Cape Province of South Africa. The Northern Cape covers an area of 372,889km^2^ with an estimated population of 1,193,780. The Province is rich in minerals and also has fertile agricultural land (Department of Environmental Affairs, 2019). Figure 1 shows the map of South Africa with air monitoring stations.

In Kimberley, the leading air pollution sources are diamond mining activities, vehicle emissions, and industrial facilities, where particulate matter (PM_2.5_) is a primary concern (Becker et al., 2024). Moreover, the annual measure of particulates (PM_10_) and sulfur dioxide (SO_2_) are the two most prevalent pollutants in South Africa (Forestry Fisheries and the Environment, 2024). Furthermore, power stations in South Africa emit different kinds of air pollutants, including Carbon Monoxide (CO), NO_2_, and sulfur dioxide (SO_2_) (Agbehadji and Obagbuwa, 2025a). Unfortunately, the activities of large-scale diamond mining companies in Kimberley impact negatively on the environment, leading to land degradation, air pollution and biodiversity loss (Charumbira and Ncube, 2022).

This study aims to develop a model for predicting multiple air pollutant concentrations including PM_2.5_, PM_10_, Ozone (O_3_), SO_2_, NO_2_, NO, and NO_x_. Additionally, meteorological factors such as wind speed (WS), ambient temperature (AT), relative humidity (RH) and solar radiation (SR) were considered in developing the predictive model. The generalized additive model (GAM) and deep learning time-series models, such as LSTM, were utilized for nonlinear relationship modeling and capturing temporal dependencies in the pollutants dataset. A causal inference model is leveraged to establish the impact of predicted air pollutants and others. Given that deep learning models have complex network structures, hybridizing with another model could increase the level of complexity, thus, the post-hoc explanation is employed to explain the feature influences on target variables and the model complexity. The post-hoc techniques, such as LIME, are gaining popularity in providing explanations on the relationship between a target and predictors, and provide feature importance to help with human understanding. Our study contributes toward developing a predictive model for air monitoring stations that seek to use an enhanced deep learning model with causal inference capability, as an alternative to legacy-based air pollutant concentration prediction systems. The sections are section 2 (literature review), section 3 (Method and material), section 4 (results), section 5 (discussion), section 6 (methodological limitations) and section 7 (conclusion).

## 2 Literature review

This section presents a review of generalized additive models, deep learning time-series models and post-hoc explanation models focusing on air pollutant concentration prediction within their air quality thresholds. It then presents a summary of models, either single or hybrid models, applicable to diverse research domains within the context of air pollution predictions and quality indexes.

### 2.1 Generalized additive model

The GAM is a model that handles the nonlinear relationship among features. GAM handle this nonlinearity through the use of link functions to map input data to their search space (Nisbet et al., 2009). By so doing, it uses probability distributions to achieve the mapping to the data distribution. This model incorporates smoothing functions (like splines) to model the relationship, thereby allowing the capture of the nonlinearity in data variables. By so doing, it offers interpretability to models. Additionally, neural networks, when deployed on GAM, can estimate the smoothing function to offer scalable models. The degree of flexibility of the GAM makes it suitable for its incorporation in other models. Jo and Kim (2023) indicated that using neural networks on GAM offers good prediction performance for multivariate time-series predictions (MTS) and also offers interpretability for neural networks (which are black-box in nature). Integrating a neural network on GAM helps estimate each feature contribution toward an accurate and explainable deep learning model (Ortega-Fernandez et al., 2024). Studies such as Lou et al. (2012) and Obster et al. (2022) also applied machine learning models to GAM to enhance accuracy. Studies have been conducted to compare the utilization of interpretability of tools like GAM and Shapley additive explanations (SHAP) among scientists and concluded that there is over-trust and misuse of these tools (Kaur et al., 2020). Given this, the merit and demerits of GAM and machine learning models were highlighted, thus discounting the notion that there is no strict trade-off between models’ performance and interpretability, thereby dispelling the idea that only black box models can achieve high performance (Kruschel et al., 2025).

Since deep learning time series models can handle nonlinear relationships as well as GAM can also handle nonlinear relationships, presenting a common approach for integration. Thus, the two-layer approach can help to improve the accuracy of models. Within a search space, the GAM can search for nonlinear transformations of both target and predictors that can result in additive model. Though GAM is a statistical tool, it provides more analytical tools for climate modeling than traditional linear models (Ravindra et al., 2019). The GAM is also very effective at handling time-series data. GAM provides a best fit for non-linear relationships between the independent variable and the predictor (Ravindra et al., 2019). However, the ease of understanding the output of the model has been a challenge when more complex nonlinear models are utilized in the prediction.

Bai et al. (2021) indicated that air quality standards are not static and need to be revised after a certain period. For instance, in 2013, China developed new air quality standards in which the GAM was used to evaluate this new standard on air pollutants such as PM_10_, SO_2_, and NO_2_. Their findings suggest that the new standard affected the health of the population (Bai et al., 2021). Barbalat et al. (2024) used the GAM to aggregate the prediction of three machine learning models, such as extreme gradient boosting, random forest and categorical boosting, to predict daily NO_2_ concentration with low error levels. In this regard, combining the predictions increases the chance of an accurate prediction over time. The GAM has been extensively applied to analyze the relationship between air pollutants, environmental factors and health-related issues (Gao et al., 2025; Fu et al., 2023; Fu et al., 2020; Fang et al., 2021; Fan et al., 2024; Gu et al., 2020). Common air particles such as PM_10_ can remain in the atmosphere for minutes or hours, whereas PM_2.5_ can remain for days or weeks.

GAM demonstrates optimal performance in capturing the nonlinear relationship between concentration O_3_ and predictor factors than the multiple linear regression model for different locations (Habeebullah, 2020). GAM is also able to disclose the varying dependencies between air pollutants (such as PM_10_ and NO_2_) and meteorological variables for different locations and seasons (Haddad and Vizakos, 2021). Table 1 presents research on the use of GAM in different research domains.

**Table 1:** Generalized additive model in air pollution.

Author	Research focus	Approach
Zhu et al. (2024)	To predict monthly average concentrations of Nitric oxide (NO).	Spatio-temporal smoothing models and machine learning prediction algorithms.
Bitz et al. (2024)	Prediction of urban ultrafine particle emission fluxes.	Method of evaluation: *R*^2^
Adamkiewicz et al. (2022)	The short-term effect of daily PM_10_, PM_2.5_ concentration, and meteorological variables on hospital data.	GAM and a random-effects meta-analysis.
Cheng et al. (2021)	Influence of weather and air pollution on the concentration change of PM_2.5_	GAM and gradient boosting machine (GBM) approaches to analyze the relationship between PM_2.5_ concentration and environmental factors.
de Asís López et al. (2024)	Location scaling model that finds the means for each variable and a correlation matrix.	GAM to scale spatial location data
Hammouda et al. (2021)	Quantification of the impact of meteorological conditions and precursor concentrations on air pollution (PM_10_ and O_3_).	GAMs
He et al. (2023)	Estimate the daily ground-level NO_2_ concentrations.	GAM to ensemble machine learning models such as random forest and extreme gradient boosting (XGBoost).
Huang et al. (2025)	To estimate and characterize the spatiotemporal distributions of elemental PM_2.5_ in Taiwan.	An ensemble machine learning approach that combines generalized additive model (GAM) with eXtreme Gradient Boosting (XGBoost). Use the Shapley additive explanations analysis to find the time-invariant features.
Lin et al. (2025)	Meteorological and traffic effects on air pollutants such as CO, NO, NO_2_, and Nitrogen oxides (NOx); and O_3_, PM_10_, and PM_2.5_, respectively.	LSTM model incorporate GAM and Bayesian network.
de Foy et al. (2024)	Interpretable machine learning tools to analyze PM_2.5_ sensor network data.	Generalized additive models.
Ravindra et al. (2019)	GAM to address the non-normal distribution of the environment and the meteorological dataset.	Generalized additive models.
Goudet et al. (2018)	Cause-effect inference.	Use of Causal Generative Neural Network (CGNN).

Despite the application of generalized additive models (GAMs), it lacks structures that offer the needed scalability when large volumes of data are required (Chang et al., 2022). GAM is more interpretable than models such as post-hoc explanations (SHAP) (Kaur et al., 2020). The hybridization of GAM with deep learning models such as GRU, BiLSTM, and 1DCNN remains a research domain that needs to be explored within the context of air pollution prediction. Therefore, applying GAM, this research provides two insights: that is model’s interpretability and performance.

### 2.2 Time-series deep learning models

Many attempts have been made to improve the interpretability of deep learning models, leading to the use of models such as neural-based GAM and post-hoc models (such as SHAP). The gradient-based approach (such as the Adam method) has been used as an approach to smooth parameters used in GAM (Dammann et al., 2025). Deep learning models have multiple layers to process input data, reveal the hidden structures in a large set of data, improve training and model’s performance (LeCun et al., 2015). Deep learning also has “Dropout” as a way to prevent overfitting in deep learning models (Srivastava et al., 2014).

Setianingrum et al. (2022) suggested the use of the Prophet Facebook model and the Bayesian Optimisation as a hyper-parameter tuner to effectively predict O_3_, SO_2_, and CO. While traditional models like autoregressive integrated moving average (ARIMA) are more accurate for predicting the parameters of PM_10_ and NO_2_. Since model performance differs across different air pollutant datasets, there is a need to continuously enhance the model’s performance. Eslami et al. (2020) indicated that the convolutional neural network (CNN) model is faster in predicting hourly O_3_ concentrations throughout the year than both LSTM and deep stacked auto-encoder (SAE). Also, the CNN predictions of daytime O_3_ concentrations were more accurate than nighttime. However, the CNN model under-predicted the daily maximum ozone concentrations, particularly during the summer, because of the non-availability of several meteorological parameters, such as cloud fraction and solar radiation, to train their model. The complexity of interpreting learning models and generalizing their outcomes leads to the use of causal models integrated into complex models such as deep learning models (Tejada-Lapuerta et al., 2025).

The literature review highlighted model hybridization for air quality prediction. Among such hybrid models include a tree-based ensemble deep learning model, where the approach used random forest, XGboost, and Light Gradient Boosting Machine (LightGBM) to predict O3 concentration independently and then combined the predicted result with a linear regression model for further predictions (Zang et al., 2021); the application of Modified Particle Swarm Optimization (MPSO) and ANFIS (Adaptive Neuro Fuzzy Inference System) to predict SO_2_ and O_3_, in which MPSO was used to train ANFIS for better prediction of SO_2_ and O_3_ (Talati et al., 2023). Also, an adaptive filter technique based on ANN has been used to predict the daily O_3_ concentrations using the meteorological dataset (Taormina et al., 2011). Again, while Sharma et al. (2024) attempt to forecast air quality using machine learning methods, they only focus on air pollutants without considering meteorological features in testing the effectiveness of air predictive models. Meteorological features have useful information on RH, AT and WS, which are location or spatial information that can be useful in air quality prediction (Agbehadji and Obagbuwa, 2024). Unfortunately, due to data limitations or uncertainties in collecting data from multiple sources (Xiong et al., 2024), some researchers may either focus only on air pollutants or meteorological data. Therefore, this could impact how models are developed, in addition to setting hyperparameters of models.

Widiputra et al. (2021) indicates that most time-series models use univariate approach for prediction, while a multivariate CNN-LSTM uses the best features from many time-series models to make predictions. Thus, the assumption is that a group of time series data from the same source tend to have a relationship and each influences the other. By using the multivariate approach on CNN-LSTM model, the CNN layer extracts the main features from time-series data, and the LSTM layer calculates the final prediction (Widiputra et al., 2021). In the multivariate approach, the input data structure accommodates multiple parallel time-series data. This model was able to predict the value of different targets. Again, a two staged model was proposed to predict multivariate time-series data in urban area (Naz et al., 2024).

Tian et al. (2023) indicates that when large volumes of data are fed into time-series models for long-term series predictions, their performance is challenged. Thus, the parallel model can greatly improve the performance of these models.

### 2.3 Post-hoc explanation

Post-hoc explanation utilized artificial intelligence models such as SHAP and local interpretable model-agnostic explanation (LIME) to provide explanations on models. Predictive ML models such as XGBoost have been noted to provide a robust approach when compared with explainable models such as SHAP (Dillon et al., 2025). Moreover, it provides some information on the relationship between input variables and their predicted variables used in explaining ML models.

Compared to existing literature, our study hybridized GAM with LSTM and xAI to help fill the gap of interpretable and transparent modeling of multiple air pollution. Furthermore, the integration of interpretable and causality into time-series model is a relevant contribution in the context of explainable AI for environmental data monitoring and analysis. In spite of LSTM techniques, GAM provides a way forward when interpretability matters as well as accuracy of prediction. Furthermore, GAM is less data hungry as compared to LSTM, which requires large volumes of data for model training.

## 3 Materials and methods

### 3.1 Proposed method

**Figure 2:**
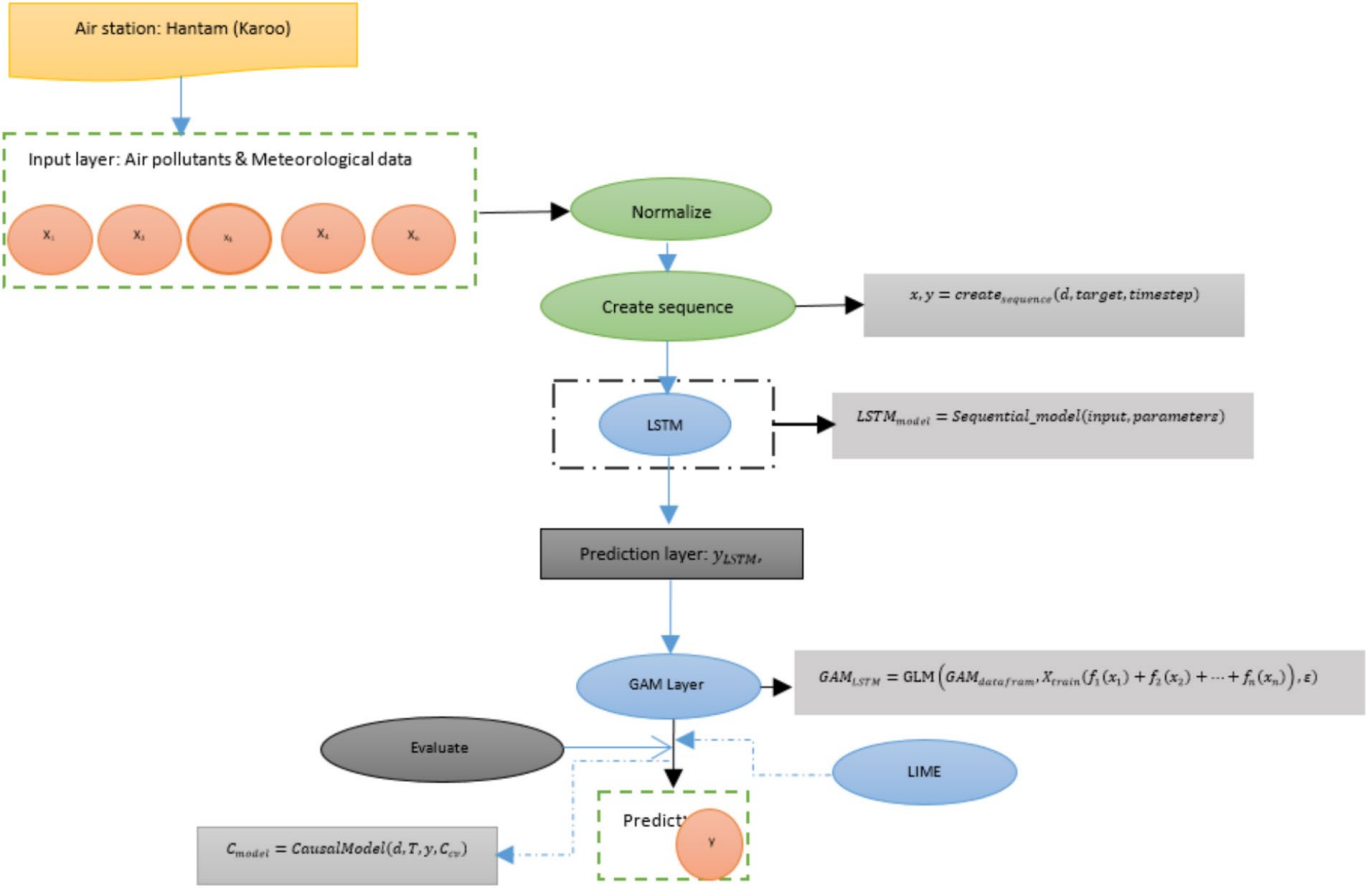
Proposed model.

In this study the objective is to propose a model for predicting multiple air pollutant concentration. The method is based on generalized additive model (GAM) and deep learning time-series models. While the time-series deep learning models can capture both nonlinear relationships and temporal dependencies, the GAM provides an initial layer that captures the nonlinear relationship in the features and the target. The aim is to improve the accuracy of the predictive model. The proposed method consists of an input layer, data preprocessing, and training Deep learning time-series model (e.g., Long short-term memory (LSTM)) layer, training a GAM layer (using deep learning predictions with features), model evaluation, predictions, and post-hoc explanation (using LIME to interpret GAM predictions). Figure 2 depicts the proposed method for this study. Integrating these approaches helps capture both temporal dynamics and their interpretable relationship between features.

### 3.2 Phases of the proposed model

Phases of the proposed model can be expressed as follows:

#### 3.2.1 Dataset

**Figure 3:**
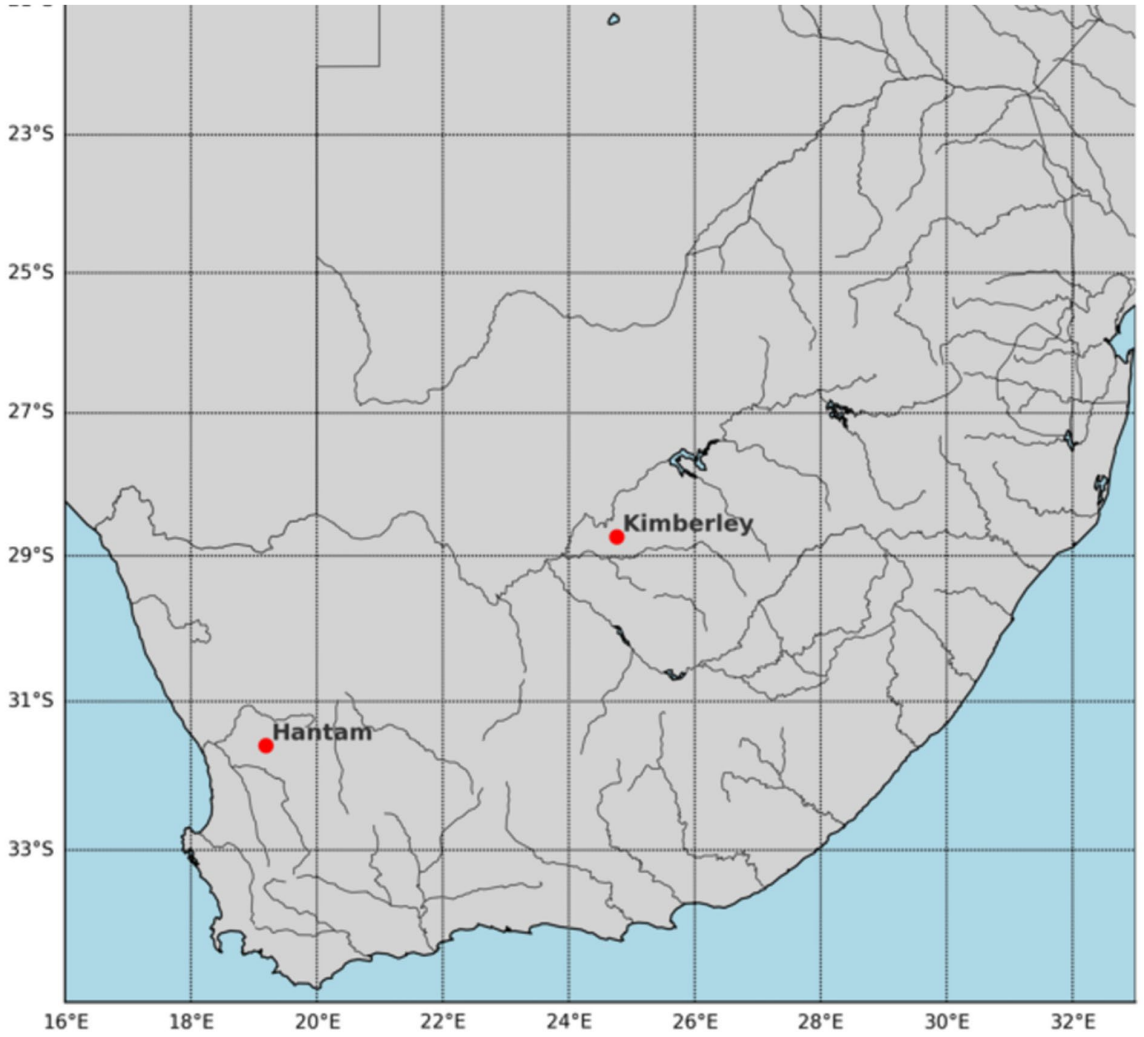
Map of South Africa showing location of Hantam and Kimberley.

This study leveraged the South African Air Quality Information System (SAAQIS) platform for recent air quality data and reports. Air pollutant data from the Northern Cape National Air Quality Indicator (NAQI) ambient monitoring network from the Hantam (Karoo) station was utilized, focusing largely on the February 2024 pollutant data, which has statistical information such as average, minimum (min) and maximum (max) range for 8-h running average. Statistical information on other features was leveraged from the February 2021 report (that is, CO and WS) and February 2023 (that is, O_3_) to augment the February 2024 statistical information. Based on this information, a dataset was synthesized for the city of Kimberley, which contained meteorological and air pollutant concentrations, thereby addressing the data limitation gap. Figure 3 is a map highlighting the location of Hantam and Kimberley, this map was obtained using Python library.

#### 3.2.2 Input layer

The synthesized dataset was generated using a random sampling method from a normal distribution, which is bounded by the statistical information like min/max values for each environmental variable and pollutant data from Hantam (Karoo) air monitoring station in Northern Cape Province in the Republic of South Africa. Data was loaded as input into the model for data preparation. The data consists of PM_2.5_, PM_10_, CO, O_3_, SO_2_, NO_2_, NO_x_, NO, WS, AT, RH and SR.

Having loaded the data, it is cleansed through data interpolation, then it is normalized and the LSTM sequence is created. Afterwards, it is split into training and test, then loaded into the LSTM model. Data normalization was achieved using the *MinMaxScaler* method. There were 6,210 instances with 12 features. After normalization, the time sequence is created using the dataset, target and time-step, which is expressed by Equation (1):


(1) $$x,y=\text{creat}e_{\text{sequence}}(\text{dataset},\text{target},\text{timestep})$$

Where *x* represents the features and *y* is the target pollutant, dataset consists of the air pollutants and the target represents what was being predicted. In this regard, the time-step and the features are input into the deep learning time-series models.

#### 3.2.3 Deep learning time-series models

The deep learning models are used to capture the temporal dependencies in the air pollutant concentrations and meteorological variables. The time-series deep learning models considered are LSTM, BiLSTM, GRU, BiGRU, 1DCNN and Transformer. The input features into this layer are of PM_2.5_, PM_10_, CO, O_3_, SO_2_, NO_2_, NO_x_, NO, WS, AT, RH and SR. These single models are hybridized with the GAM model to provide a hybrid model for the prediction of CO_2_ concentration. These deep learning (DL) models are trained on the original input dataset, and the trained model is used for prediction. DL model is expressed in Equations (2–4).


(2) $$\mathrm{LST}M_{\text{model}}=\text{Sequential}\_\text{model}(\text{input},\text{parameters})$$

(3) $$\mathrm{LST}M_{\text{model}}=\mathrm{LST}M_{\text{model}}(x_{\text{train}},y_{\text{train}})$$

(4) $$\mathrm{LST}M_{\text{predict}}=\mathrm{LST}M_{\text{model}}(x_{\text{test}})$$

Where $\mathrm{LST}M_{\text{model}}$ represents the LSTM model and Sequential_model () represents a deep learning library with input and parameters.

#### 3.2.4 Generalized additive model layer

After training the deep learning model to make the predictions, the GAM was used as a post-processing approach to model the relationship between the predictions and actual data, thus allowing for model interpretability and refinement of the predictions. Thus, the generalized additive model is flexible for modeling nonlinear relationships between predictors and the target pollutant. The GAM could be generally expressed in Equations (5, 6):


(5) $$y=\beta_{0}+f_{1}(x_{1})+f_{2}(x_{2})+\ldots +f_{n}(x_{n})+\varepsilon$$

Thus,


(6) $$y_{i}=\beta_{0}+\sum_{i=1}^{n}f(x_{i})+\varepsilon_{i}$$

Where *y* is the predicted air pollutant, $f(x_{i})$ is the nonlinear function (*f_1_,…,f_n_* are the smooth functions representing the effect of each predictor like *x_1_, x_2_,…,x_n_*), *β_0_* is the intercept term, and epsilon (*ϵ*) is the error term.

The generalized additive model has a smoothing function that explains each feature’s relation with the target pollutant. For instance, features such as PM_2.5_, PM_10_, SO_2_, O_3_, NO_2_, NOx, WS, AT, RH and SR are the independent variables whereas the target pollutant like NO concentration is the dependent variable which could be explained with GAM in Equation (7).


(7) $$\begin{array}{l} \mathrm{NO}=\beta_{0}+f_{1}\;(PM_{2.5})+f_{2}\;(PM_{10})+f_{3}\;(SO_{2})+f_{4}\;(O_{3})+\\ f_{5}\;(NO_{2})+f_{6}\;(\mathrm{NOx})+f_{7}\;(\mathrm{WS})+f_{8}\;(\mathrm{RH})+\\ f_{9}\;(\mathrm{AT})+f_{10}\;(\mathrm{SR})+\in \end{array}$$

Again, to predict SO_2_, the GAM is expressed by Equation (8):


(8) $$\begin{array}{l} SO_{2}=\beta_{0}+f_{1}\;(PM_{2.5})+f_{2}\;(PM_{10})+f_{3}\;({\mathrm{NO}}_{x})+f_{4}\;(O_{3})+\\ f_{5}\;(NO_{2})+f_{6}\;(\mathrm{NO})+f_{7}\;(\mathrm{WS})+f_{8}\;(\mathrm{RH})+\\ f_{9}\;(\mathrm{AT})+f_{10}\;(\mathrm{SR})+\in \end{array}$$

Furthermore, the remaining air pollutant concentrations (PM_2.5_, PM_10_, O_3_, NO_2_, NO_x_) are predicted subsequently to understand the correlation between air pollutant features and a target pollutant. The integration of the LSTM model with GAM was achieved using dataframe, expressed as Equation (9):


(9) $$\mathrm{GA}M_{\text{datafram}}=(y_{\text{test}},\mathrm{LST}M_{\text{predict}})$$

Thus, $\mathrm{LST}M_{\text{predict}}$ represent the predictions from the LSTM model and $y_{\text{test}}$ is the test dataset, $\mathrm{GA}M_{\text{datafram}}$ is the dataframe. Generally, the LSTM-GAM model is expressed by Equation (10):


(10) $$\mathrm{GA}M_{\text{LSTM}}=\mathrm{GLM}(\begin{array}{l} \mathrm{GA}M_{\text{datafram}},X_{\text{train}}(f_{1}(x_{1})+\\ f_{2}(x_{2})+\ldots +f_{n}(x_{n}) \end{array}),\varepsilon )$$

Where $f_{1}(x_{1,})$ etc. represent the feature matrix of the training set, ε represents the error term, GLM () is the function to implement the generalized additive model which is important from GLM library, and $\mathrm{GA}M_{\text{datafram}}$ represents the target air pollutant that is the intercept β_0_. Equation (11) was used to fit the model, and Equation (12) was used for prediction.


(11) $$y_{\mathrm{GAM}\_\text{predict}}=\mathrm{GA}M_{\text{LSTM}}.\mathrm{fit}()$$

(12) $$y_{\mathrm{GA}M_{\text{final}}}=y_{\mathrm{GAM}\_\text{predict}}.\text{predict}(x)$$

Where $y_{\mathrm{GA}M_{\text{final}}}$ represents the final prediction from the LSTM-GAM. The residual or error term ε was normally distributed using the Gaussian function. The $y_{\mathrm{GA}M_{\text{final}}}$ model was used by the LIME model for interpretation, which is expressed as Equation (13).


(13) $$A_{\text{LIME}}=f(y_{\mathrm{GA}M_{\text{final}}})$$

Where $A_{\text{LIME}}$ represents the LIME explainer, $f(y_{\mathrm{GA}M_{\text{final}}})$ is the function that explains the final predictions to display the output for visualization and causal inference analysis.

#### 3.2.5 Model evaluation metrics

The models were evaluated with mean squared error (MSE), root mean squared error (RMSE) and mean absolute error (MAE). The MSE is expressed in Equation (14).


(14) $$\mathrm{MSE}=\frac{1}{N}\sum_{i=1}^{N}{\left(y_{i}-\bar{y}\right)}^{2}$$

Where $\bar{y}$ is the predicted value from the model, $y_{i}$ is the actual value of the target pollutant. The interpretation is that a lower MSE value means a better model, and a higher MSE suggest the model is worse. MSE equal to zero means the model’s prediction is perfect. The RMSE is expressed in Equation (15).


(15) $$\text{RMSE}=\sqrt{\mathrm{MSE}}$$

The MAE is expressed by Equation (16).


(16) $$\mathrm{MAE}=\frac{1}{N}\sum_{i=1}^{N}(y_{i}-\bar{y})$$

Where $\bar{y}$ is the predicted value of the target pollutant, $y_{i}$ is the actual value of the target pollutant, and N represents the number of data points.

One of the key methods to evaluate a model is the differences between the actual value and prediction, which should be lower to suggest better prediction, that is, the predicted variable has a value closer to the observed value (Wattal and Singh, 2021). Tian et al. (2023) indicates that when large volumes of data are fed into time-series models for a long-range prediction, their performance is challenged, thus, it is imperative to understand how the model performs to suggest future enhancements.

#### 3.2.6 Post-hoc explanation

Post-hoc explanation explains a decision or prediction of a model. The goal is to interpret the inner workings or the output of a complex model in a simple, human-understandable manner, like linear regression. Thus, it explains how to understand the reason models make certain decisions using an M-day time-step of previous data. These explanations are needed to increase transparency, ensure fairness, improve trust, and satisfy regulatory. In this study, LIME was used to explain the contribution or correlation of each feature in the final prediction. Thus, feature importance and their weighted values are displayed for interpretation. In this regard, to explain the prediction $f(x_{n})$ to a complex model f at each point $x_{n}$ , the simple linear regression for LIME can be mathematically expressed to approximate f to an interpretable model g within the local neighboring $x_{n}$ variables by Equation (17).


(17) $$g(x)=\arg\min_{g\in G}L(f,g,\pi_{x})+\delta (g)$$

Where the components f is the complex model, g is the interpretable model (that is the linear regression); $L(f,g,\pi_{x})$ represent the local loss function which measure how well g can approximate f in the neighboring variables x ; $\pi_{x}(z)$ explains how close sampled points z is to the original point x ; and δ(g) is the complexity penalty weight to ensure a simple model. Equations (18–20) represents the mathematical expression.


(18) $$f(x)=\mathrm{LST}M_{\text{prediction}}(x)$$

(19) $$g(x)=w_{0}+\sum_{i=1}^{n}w_{i}x_{i}$$

(20) $$\pi_{x}=\exp(-\frac{D(x,x\prime )}{\sigma^{2}})$$

Thus, kernel function computes *D*, which is the distance between x and x′ ; variance σ between x and x′ ; *n* is the number of features in the matrix; $w_{0}$ is the baseline intercept where the coefficients $w_{i}$ are the relative importance of each feature.

#### 3.2.7 Causal inference analysis

The causal inference model was leveraged to understand the effect or impact of target air pollutants on predictor pollutants. In this regard, LIME helps to display the features according to their relative importance. The most relevant feature has a higher weighted value, which is then mapped to the target air pollutant using the causal inference model to understand the impact. The other less relevant features are included in the causal inference model as confounding variables (common causes) for a comprehensive mapping. Thus, the causal inference model $C_{\text{model}}\;$ can be expressed by Equation (21):


(21) $$C_{\text{model}}=\text{CausalModel}(d,T,y,C_{\mathrm{cv}})$$

Where *d* represents dataset, *T* is the treatment (target pollutant, e.g., $SO_{2}$ ), *y* is the outcome (e.g., ${\mathrm{NO}}_{x}$ ), and $C_{\mathrm{cv}}$ is the common causes, where $C_{\mathrm{cv}}\in [\;NO_{2},\mathrm{NO},O_{3},PM_{2.5},PM_{10},\text{WSpeed},\mathrm{AT},\mathrm{RH},\mathrm{SR}]$ .

The causal effect is estimated using Equation (22):


(22) $$Y=\mu +\mathrm{\gamma T}+\sum_{i=1}^{n}\beta_{i}X_{i}+\in$$

Where γ is the approximated or estimated causal effect of *T* on *Y,* $\beta_{i}$ is the effect of the confounders $X_{i}$ , ∈ is the noise (that is, random error term), μ is the intercept. The terms are μ intercept term, causal effect treatment term is γT , and $\sum_{i=1}^{n}\beta_{i}X_{i}$ is the effect of confounder terms, and error term is ∈. For each observation y and predicted y, the error term ∈ is expressed by $\in_{i}=y_{i}-Y_{i}.$

### 3.3 Model parameter description

Table 2 presents the parameter description of the proposed model. The proposed and comparative models were implemented in Python 3.10 due to its programming flexibility, using an Intel Core i3 processor. The relevant deep learning libraries were imported to develop the model. Dropout and regularization was used to avoid overfitting in the deep learning models. Our model training procedure focused on 80% data for training and 20% for testing, while the use of 80% allows sufficient data use for training.

**Table 2:** Hyperparameter tuning.

Model	Parameter value
LSTM	Hidden units = 50
	Batch size = 32
Dense	1
Optimizer	“adam”
Epoch	100
Activation function	“relu”
Error ( ε )	Gaussian function
M-day time-step	M: 5, 10

The parameter values followed the study by Zhang et al. (2023) on hidden units and epoch, and Hwang et al. (2024) on batch size. Optimizer used is “adam” with relu as the activation function of our proposed model. Furthermore, the random seed control strategy utilized numpy among others which ensures model’s reproducibility during models execution. After completing the 100 epoch, the final model was selected based on the best validation performance.

## 4 Results

### 4.1 Models’ prediction over M-day time-step

During the experiment, M-day time-step was considered where M represents the number of previous days data used to train the deep learning time-series models. Table 3 presents the evaluation results of the models prediction with 5-day time-step previous data.

**Table 3:** Air pollutants predictions with 5-day time-step.

Model	Predict NO_x_			Predict CO			PredictPM_2.5_			Predict PM_10_		
	MSE	RMSE	MAE	MSE	RMSE	MAE	MSE	RMSE	MAE	MSE	RMSE	MAE
LSTM	**0.340**	**0.583**	**0.736**	0.0296	0.1722	0.1354	1.152	1.0733	0.923	1.571	1.2534	0.964
BiLSTM	1.625	1.275	0.807	0.0366	0.1914	0.1513	1.441	1.2004	0.123	1.818	1.3483	1.036
BiGRU	1.487	1.219	0.762	0.0372	0.1929	0.1521	1.424	1.1933	1.078	1.923	1.3867	1.073
1DCNN	1.402	1.184	0.694	0.0253	0.1589	0.1287	0.960	0.9798	0.658	1.297	1.1389	0.923
LSTM-GAM-xAI	0.990	0.995	0.549	**0.0184**	**0.1355**	**0.1100**	**0.703**	**0.8385**	**0.502**	**0.954**	**0.9767**	**0.794**
Random Forest	1.394	1.181	0.673	0.1376	0.3,709	0.1130	0.783	0.8849	0.538	1.004	1.0020	0.841
XGBoost	1.502	1.226	0.680	0.0218	0.1477	0.1173	0.910	0.9539	0.603	1.147	1.0710	0.887

Model	Predict O_3_			Predict SO_2_			Predict NO			Predict NO_2_		
	MSE	RMSE	MAE	MSE	RMSE	MAE	MSE	RMSE	MAE	MSE	RMSE	MAE
LSTM	379.349	19.4769	16.632	0.607	0.7791	0.862	1.217	1.1032	0.886	0.886	0.9413	0.732
BiLSTM	420.932	20.5166	18.569	0.806	0.8978	0.965	1.639	1.2802	0.999	0.980	0.9899	0.764
BiGRU	414.866	20.3683	18.231	0.796	0.8922	0.969	1.581	1.2574	1.009	0.999	0.9995	0.770
1DCNN	289.449	17.0132	16.324	0.621	0.7880	0.832	1.101	1.0493	0.8534	1.002	1.0010	0.923
LSTM-GAM-xAI	**198.787**	**14.0992**	**12.732**	**0.416**	**0.6450**	**0.501**	**0.786**	**0.8866**	**0.9051**	**0.658**	**0.8112**	**0.432**
Random Forest	219.103	14.8021	13.342	0.427	0.6535	0.556	0.867	0.9311	0.754	0.653	0.8081	0.489
XGBoost	245.787	15.6776	14.321	0.497	0.7050	0.801	0.990	0.9950	0.8041	0.759	0.8712	0.472

*Bold values indicate the best metric values for the respective model.

Table 3 shows the 5-day time-step previous of the models to predict air pollutants such as NO_x_, PM_2.5_, PM_10_, O_3_, SO_2_, CO, NO and NO_2_. These predictions help in analyzing the predictive performance of each model and comparing it with the proposed model. For instance, in predicting the NOx, it was observed that LSTM, BiLSTM, BiGRU, IDCNN yielded the following MSE values 0.340, 1.625, 1.487, 1.402 and 0.990, respectively, for the deep learning time series models. It emerged that LSTM generated the lowest MSE value of 0.340, while the proposed LSTM-GAM-xAI guaranteed the second least MSE value of 0.990. Comparatively, LSTM-GAM-xAI performed better as it consistently maintained the lowest MSE value in all the air pollutant predictions using the deep learning time-series models.

Again, the two most common machine learning models, such as Random Forest (RF) and XGBoost were also compared with the comparative models. It can be observed that the MSE value for RF is 1.394, and XGBoost guaranteed 1.502. Based on these findings, the proposed LSTM-GAM-xAI guaranteed the least MSE value than the comparative ML models for predicting NO_2_ concentration. Furthermore, the proposed LSTM-GAM-xAI guaranteed the best performance across all the air pollutant predictions.

Table 4 shows the statistical information of the LSTM-GAM-xAI model on SO2 predictions for 5-day time-step. It can be observed that, there were 1,237 observation on features. Again, NO_2_ had coefficient (coef) of 0.0170 with a constant term $\beta_{0}$ of 1.3411, standard error (std err) of 0.178. Solar radiation had coef of 2.35×10^−05^ which is the least among the coefficient values.

**Table 4:** LSTM-GAM-xAI statistical information on SO_2_ concentration with 5-day time-step.

Variable	Coef	Std. err.	z	*p* > |z|	[0.025	0.975]
Const	1.341	0.178	7.519	0.000	0.992	1.691
NO_2_	0.0170	0.023	0.748	0.455	−0.028	0.062
NO	0.0002	0.021	0.011	0.991	−0.041	0.041
NOx	0.0133	0.019	0.718	0.473	−0.023	0.050
CO	−0.0926	0.136	−0.680	0.496	−0.359	0.174
O_3_	0.0015	0.001	1.155	0.248	−0.001	0.004
PM_2.5_	0.0137	0.022	0.625	0.532	−0.029	0.057
PM_10_	0.0007	0.019	0.036	0.971	−0.036	0.038
WS	−0.0001	0.002	−0.061	0.951	−0.003	0.003
AT	0.0020	0.004	0.502	0.616	−0.006	0.010
RH	0.0022	0.001	1.770	0.077	−0.000	0.005
SR	2.35×10^−05^	9.74×10^−05^	0.241	0.809	−0.00	0.000

Based on Table 4, it can be observed that in predicting the target pollutant (SO2), the constant term $(\beta_{0}=1.3411),$ NO_2_ contributed coef of −0.0170, NO (0.0002), NOx (0.0133), CO (−0.0925), O_3_ (0.0015), PM_2.5_ (0.0137), PM_10_ (0.0007), WS (−0.0001), AT (0.0020), RH (0.0022) and SR (2.35e-05). Thus, the general expression can be deduced within the confidence interval (0.025 and 0.975) as follows:


$$\begin{array}{l} SO_{2}=\beta_{0}-0.0170\cdot \mathrm{NO}2+0.0002\cdot \mathrm{NO}+0.0133\cdot \mathrm{NOx}-\\ 0.0925\cdot \mathrm{CO}+0.0015\cdot O3+0.0137\cdot \mathrm{PM}2.5+\\ 0.0007\cdot \mathrm{PM}10-0.0001\cdot \mathrm{WS}+0.0020\cdot \mathrm{AT}+\\ 0.0022\cdot \mathrm{RH}+(2.35e-05)\cdot \mathrm{SR} \end{array}$$

Furthermore, Appendix A1 present the detailed statistical information of air pollutants on the 5-day time-step of the previous data considered in this study.

Having experimented the 5-day time-step, further experiment was conducted using 10-day time-step previous data which aimed to further asses the performance of the models. Table 5 shows the 10-day time-step prediction of air pollutants.

**Table 5:** Air pollutants predictions with 10-day time-step.

Model	Predict NO_x_			Predict CO			PredictPM_2.5_			Predict PM_10_		
	MSE	RMSE	MAE	MSE	RMSE	MAE	MSE	RMSE	MAE	MSE	RMSE	MAE
LSTM	1.426	1.1942	0.711	0.0303	0.1739	0.1330	0.976	0.988	0.531	1.139	1.0672	0.957
BiLSTM	1.432	1.1967	0.762	0.0341	0.1846	0.1412	1.171	1.082	0.602	1.350	1.1619	1.058
BiGRU	1.493	1.2219	0.782	0.0339	0.1842	0.1419	1.304	1.142	0.621	1.471	1.2128	1.114
1DCNN	1.577	1.2558	0.802	0.0281	0.1677	0.1316	0.964	0.982	0.562	1.039	1.0193	0.980
LSTM-GAM-xAI	**0.960**	0.9798	0.509	0.0300	0.1731	0.1350	**0.725**	0.852	0.421	**0.756**	0.8695	0.608
Random Forest	1.394	1.1807	0.673	**0.0189**	0.1376	0.1130	0.754	0.868	0.523	0.814	0.9022	0.841
XGBoost	1.502	1.2256	0.680	0.0218	0.1477	0.1173	0.871	0.933	0.612	0.928	0.9633	0.887

Model	Predict O_3_			Predict SO_2_			Predict NO			Predict NO_2_		
	MSE	RMSE	MAE	MSE	RMSE	MAE	MSE	RMSE	MAE	MSE	RMSE	MAE
LSTM	1.314	1.1463	0.716	1.161	1.0775	0.732	1.365	1.168	0.830	0.897	0.947	0.526
BiLSTM	1.630	1.2767	0.796	1.355	1.1640	0.731	1,503	38.769	35.841	0.934	0.966	0.600
BiGRU	1.627	1.2755	0.792	1.485	1.2186	0.801	1.610	1.269	1.002	0.917	0.958	0.600
1DCNN	1.327	1.1520	0.631	1.090	1.0440	0.720	1.252	1.119	0.923	1.031	1.015	0.673
LSTM-GAM-xAI	**0.930**	0.9644	0.531	**0.782**	0.8843	0.602	**0.784**	0.885	0.632	**0.660**	0.812	0.377
Random Forest	1.032	1.0159	0.732	0.845	0.9192	0.712	0.867	0.931	0.754	0.653	0.808	0.452
XGBoost	1.182	1.0872	0.830	1.003	1.0015	0.753	0.990	0.995	0.789	0.759	0.8712	0.471

*Bold values indicate the best metric values for the respective model.

Based on Table 5, it is generally observed that LSTM-GAM-xAI has the best MSE values, in terms of the least MSE value, across the entire prediction of air pollutants. These results suggest that the proposed model performs well given the 10-day time-step previous data.

Table 6 shows the LSTM-GAM-xAI model’s statistical information for the 10-day time-step (e.g., for SO_2_ concentration), whereas further statistical information for the remaining air pollutants are shown in Appendix A2. The statistical information includes *coef, std err, z* and *p-value*. Where *coef* represents the estimated coefficient for the predictor pollutants, which is the effect size of how much the outcome changes with an increase in a feature when other features are constant; whereas *std* err is the standard error, *z*-score and *p*-value.

**Table 6:** LSTM-GAM-xAI statistical information on SO_2_ concentration with 10-day time-step.

Variable	Coef	Std. err.	z	*p* > |z|	[0.025	0.975]
NO_2_	−0.0178	0.043	−0.408	0.684	−0.101	0.067
NO	14.7005	94.101	0.156	0.876	−169.725	199.144
NOx	−0.0441	0.026	−1.694	0.090	−0.095	0.007
CO	−0.0197	0.051	−0.387	0.699	−0.120	0.080
O_3_	0.0250	0.026	0.954	0.340	−0.026	0.076
PM_2.5_	−0.0452	0.030	−1.522	0.128	−0.103	0.013
PM_10_	−0.0442	0.029	−1.516	0.129	−0.101	0.013
WS	−0.0100	0.025	−0.397	0.691	−0.059	0.039
AT	−0.0184	0.025	−0.727	0.467	−0.068	0.031
RH	0.0317	0.025	1.251	0.211	−0.018	0.081
SR	0.0521	0.025	2.060	0.039	−0.003	0.102

Based on Table 6, it can be observed that in predicting only SO_2_ concentration, NO_2_ had *coef* value of −0.0175, NO (14.7095), NOx (−0.0441), CO (−0.0197), O_3_ (0.0250), PM_2.5_ (−0.0452), PM_10_ (−0.0442), WS (−0.0100), AT (−0.0184), RH (0.0317) and SR (0.0521). In view of this, a general expression can be deduced within the confidence interval (0.025 and 0.975) as follows:


$$\begin{array}{l} SO_{2}=\beta_{0}-0.0175\cdot NO_{2}+14.7095\cdot \mathrm{NO}-0.0441\cdot {\mathrm{NO}}_{x}+\\ 0.0250\cdot O_{3}-0.0452\cdot PM_{2.5}-0.0442\cdot PM_{10}-\\ 0.0100\cdot \mathrm{WS}-0.0184\cdot \mathrm{AT}+0.0317\cdot \mathrm{RH}+0.0521\cdot \mathrm{SR} \end{array}$$

### 4.2 Explainable AI: LIME analysis on LSTM-GAM-xAI model

This section shows the LIME analysis results for only SO_2_ concentration within the 5-day time-step and 10-day time-step. While the detail LIME analysis results for the remaining air pollutants are presented in Appendices A3, A4.

**Figure 4:**
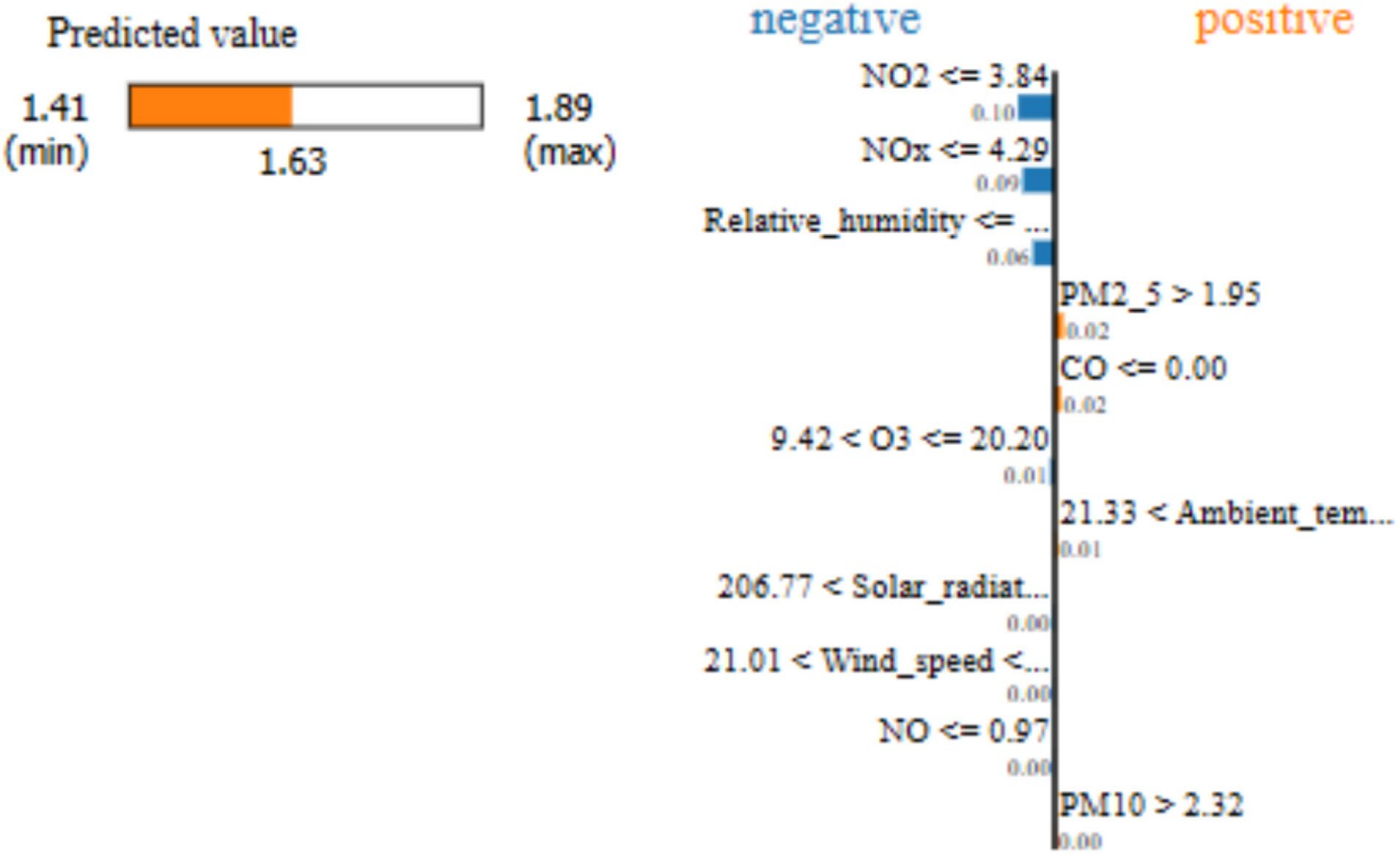
LSTM-GAM-xAI model’s LIME explanation of SO_2_ concentration with a 5-day time-step.

Figure 4 shows the LIME analysis results of LSTM-GAM-xAI model focusing on SO_2_ concentration within the 5-day time-step. It can be observed that the predicted value lies between min (1.41) and max (1.89) as shown on the horizontal bar, where the intermediate value of 1.63 is the predicted value of the LSTM-GAM-xAI model for the specific instance being explained. Furthermore, the vertical line separates the features into positive and negative ranges. In this regard, features that are located on the positive side such as PM_2.5_, CO, AT, and PM_10_ were the features pushing the prediction toward a 1.63 value.

Further LIME results of SO_2_ concentration in presented in Table 7, focusing on 5-day time-step previous using the LSTM-GAM-xAI model.

**Table 7:** LSTM-GAM-xAI model’s LIME explanation for SO_2_ prediction with 5-day time-step.

Features	Importance
NO_2_ ≤ 3.84	−0.10027
NOx ≤ 4.29	−0.08765
RH ≤ 38.48	−0.05968
PM_2.5_ > 1.95	0.02334
CO ≤ 0.00	0.01678
9.42 < O_3_ ≤ 20.20	−0.01244
21.33 < AT ≤ 24.39	0.00563
206.77 < SR ≤ 343.39	−0.00426
21.01 < WS ≤ 28.57	−0.00263
NO ≤ 0.97	−0.00214
PM_10_ > 2.32	0.00189

From Table 7, it can be observed that some features recorded negative importance value, such as NO_2_, NOx, RH, etc. Other pollutants recorded positive importance value, such as PM_2.5_, CO, AT and PM_10_ in their respective threshold values. “NO_2_ ≤ 3.84” had an importance value of −0.100271, and “PM_2.5_ > 1.95” had an importance value of 0.0233407. Where, “PM_2.5_ > 1.95” means the PM_2.5_ had a threshold value of 1.95 and it contributes positively, a value of 0.0233407, to the prediction of SO_2_. Thus, higher PM_2.5_ increases the prediction of SO_2,_ and though the effect is positive, it is relatively weak compared to NO_2_ influence on SO_2_. Thus, it can be indicated that NO_2_ contributed negatively in predicting SO_2_ and therefore contributed to pulling down the prediction relative to the local mean. On the other hand, PM_2.5_ had a value of 0.0233407, which suggests that PM_2.5_ contribute positively to the prediction and therefore pushes the prediction up. Comparatively, PM_2.5_ contribute a better positive value as compared with CO (0.0167), PM_10_ (0.00188). Thus, the simple linear model of LIME can be finally modeled as:


$$\begin{array}{l} g_{\text{LIME}\_SO_{2}}=w_{0}-0.10027(NO_{2}\leq 3.84)-0.088({\mathrm{NO}}_{x}\leq 4.29)-\\ 0.060(\mathrm{RH}\leq 38.48)+0.023(PM_{2.5}>1.95)+\\ 0.017(\mathrm{CO}\leq 0.00)-0.012(9.42<O_{3}\leq 20.20)+\ldots + \end{array}$$

**Figure 5:**
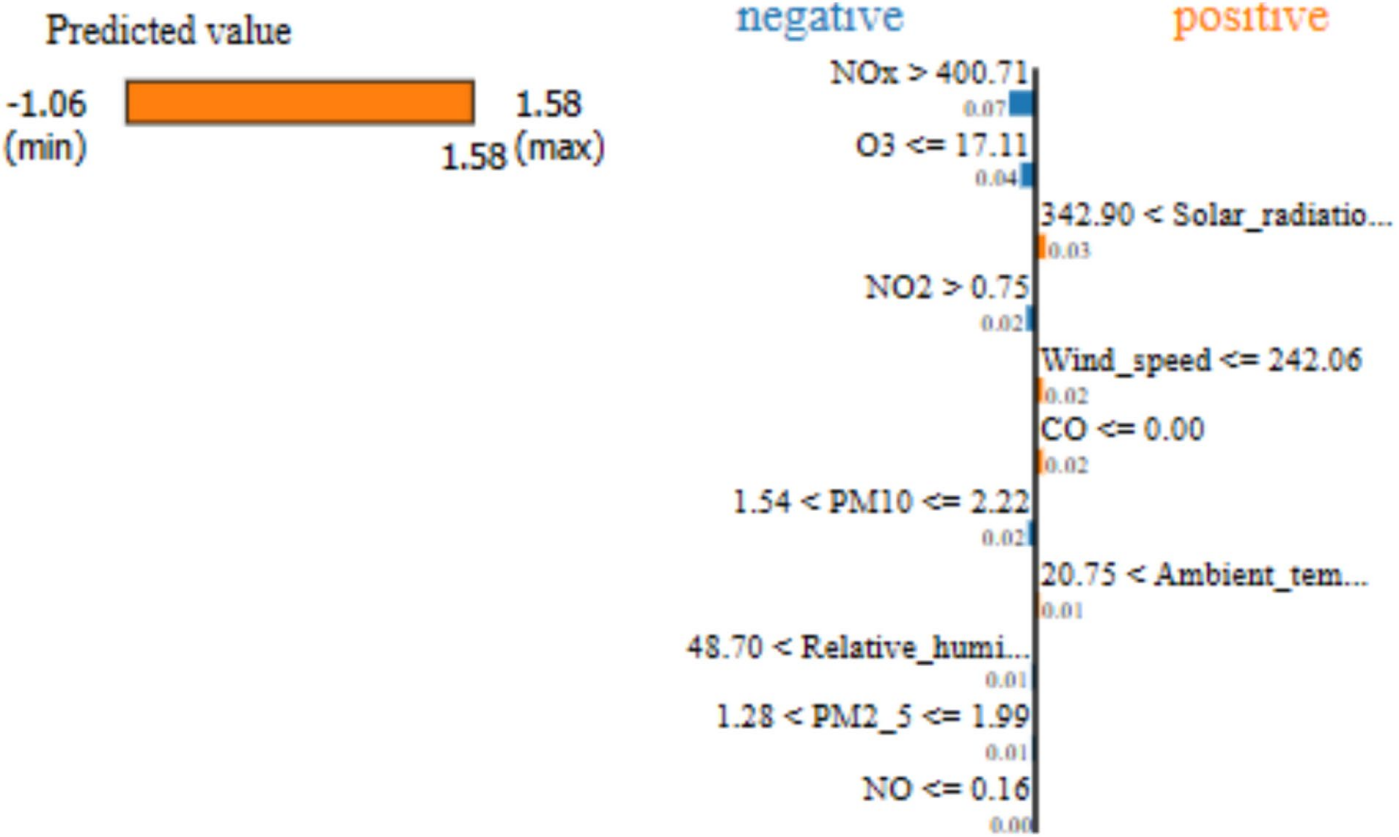
LSTM-GAM-xAI LIME explanation for SO_2_ concentration prediction with 10-day time-step.

In terms of the 10-day time-step to predict SO_2_ concentration with the LSTM-GAM-xAI model, the LIME analysis result is presented in Figure 5. It can be observed that “NOx > 400.71” has a feature value approximately −0.07, followed by “O3 ≤ 17.11” with a feature value of approx. −0.04. These results suggest that while using the 10-day time-step, previous to predict SO_2_ concentration NOx with its threshold value (>400.71) contributed negatively (−0.07) to the prediction of SO_2_. Similarly, O_3_ with its threshold ( ≤ 17.11) also contributed negatively (−0.04) or pulled down the SO_2_ concentration. On the other hand, SR ranged between (“342.90 < SR ≤ 343.59”) and contributed positively (0.02889) to the prediction of SO_2_ concentration. Again, SR was the highest positive feature value among the features recorded during the LIME explanation. Furthermore, the predicted value between the min (−1.06) and max (1.58) is 1.58 as indicated on the yellow bar. This suggests that features that contribute to pushing up the SO_2_ are SR, WS, CO, and AT. On the other hand, the features that contribute negatively or push down the prediction are NOx, O_3_, NO_2_, PM_10_, RH and PM_2.5_.

Furthermore, Table 8 shows the LSTM-GAM-xAI aspect of LIME explanation showing feature importance values for the air pollutants that contributed in SO_2_ concentration with 10-day time-step.

**Table 8:** LSTM-GAM-xAI LIME feature importance for SO_2_ prediction with 10-day time-step.

Feature	Importance
NOx > 400.71	−0.07384
O_3_ ≤ 17.11	−0.04122
342.90 < SR ≤ 343.59	0.02889
NO_2_ > 0.75	−0.02486
WS ≤ 242.06	0.02117
CO ≤ 0.00	0.02113
1.54 < PM_10_ ≤ 2.22	−0.02086
20.75 < AT ≤ 21.48	0.01126
48.70 < RH ≤ 49.39	−0.01061
1.28 < PM_2.5_ ≤ 1.99	−0.01011
NO ≤ 0.16	0.00000

### 4.3 Causal inference model

Table 9 presents the causal inference model, which explains the effect or impact of the target pollutant on another. These pollutant features are the features with high relative importance considered as the outcome. Again, other features, with less importance values, were considered confounders in the causal inference model. These features were obtained from the LIME explanation and mapped to the target pollutant. Table 9 presents the causal inference analysis using the 5-day time-step. The analysis is based on components such as estimated effect, new effect and *p*-value. SO_2_, NO, PM_2.5_, PM_10_, O_3_ and NO_2_ generated estimated effect as 0.002937, 0.008082, −0.004943, −0.003184, −0.003184 and −0.005352, respectively.

**Table 9:** Causal inferences analysis with 5-day time-step.

Target pollutant	Estimated effect	New effect	*p*-value	Outcome
SO_2_	0.002937	0.0029055	0.92	NO_2_
NO	0.008082	0.0080740	0.90	NO_x_
NO_x_	−0.003280	−0.0032964	0.88	NO_2_
PM_2.5_	−0.004943	−0.0049681	0.94	NO_x_
PM_10_	−0.003184	−0.0031820	0.96	NO_x_
O_3_	−0.003184	−0.0031741	0.89	NO_2_
CO	−0.005460	−0.0054680	0.98	NO_2_
NO_2_	−0.005352	−0.0053696	0.98	NO_x_

## 5 Discussion

The experiment results demonstrate different results to help understand the performance of the models (DL and ML) for the air pollutants prediction for the 5-day and 10-day time-steps. Common air pollutants considered are NOx, PM_2.5_, PM_10_, O_3_, SO_2_, NO and NO_2_ with meteorological features. The results indicate that the proposed model demonstrated good performance using the MSE value for 5-day and 10-day time steps. For instance, in predicting SO_2_ for the 10-day time-step, the performances are LSTM-GAM-xAI (0.782), LSTM (1.161), BiLSTM (1.355), BiGRU (1.485), 1DCNN (1.090), Random Forest (0.845), and XGBoost (1.003). Again, in predicting SO_2_ for 5-day time-step generated the following LSTM-GAM-xAI (0.416), LSTM (0.607), BiLSTM (0.806), BiGRU (0.796), 1DCNN (0.621), Random Forest (0.427), and XGBoost (0.497). These findings suggest that in predicting the hourly concentration level of the air pollutants, the proposed model demonstrated good performance against the comparative models. Huang et al. (2025) combine GAM with eXtreme Gradient Boosting (XGBoost) to predict PM_2.5_ concentration in Taiwan with SHAP explanation. Furthermore, He et al. (2023) estimated NO_2_ concentration with GAM combined with XGBoost. Cheng et al. (2021) also applied GAM combined with gradient boosting machine (GBM) to determine the relationship between PM_2.5_ concentration and environmental factors. In view of this empirical research, GAM has been applied to a machine learning model for air pollutant analysis. Our research also extends the context of research by using the GAM with LSTM and post-hoc explainable artificial intelligence model to provide more explanation on models such as DL and ML models, which are used to predict common air pollutants such as PM_2.5_, PM_10_, SO_2_, O_3_, NO, NOx, NO_2_ using different time-steps.

LIME explanation for SO_2_ concentration prediction with a 5-day time-step generated “NO_2_ ≤ 3.84” with importance value −0.10027, “NO_x_ ≤ 4.29” with importance value −0.08764, “RH ≤ 38.48” with importance value −0.05968, etc. (Table 7). Whereas, GAM produced NO_2_ with a coefficient (0.0170) followed by NO (0.0002), NOx (0.0133), CO (−0.0925), O_3_ (0.0015), etc. (Table 4). It can be observed that the explanation from GAM and LIME placed NO_2_ on top of the list of contributing factors in the prediction of SO_2_ concentration. These results suggest that on one hand, using LIME, NO_2_ contributed −0.10027 to SO_2_ prediction. On the other hand, with GAM explanations, NO_2_ contributed 0.0170 to the SO_2_ prediction. These results indicate that NO_2_ pushed up the SO_2_ prediction by approximately 0.02 in GAM, while NO_2_ reduced the SO_2_ prediction by approximately 0.10 in LIME explanation.

The LIME explanation provided the relevant features for the causal inference analysis. The causal effect generated p-value as SO_2_ (0.92), NO (0.90), NOx (0.88), PM_2.5_ (0.94), PM_10_ (0.96), O_3_ (0.89) and NO_2_ (0.98) respectively (Table 9). For instance, in predicting NO concentration, the estimated effect was 0.008082, new effect (0.0080740), p-value (0.90) and outcome (NOx), having confounders as PM_2.5_, PM_10_, SO_2_, O_3_, NO, NOx, NO_2,_ RH, AT (Table 8). It is imperative to note that having two air pollutants moving together does not necessarily mean that one variable may have caused another air pollutant to occur. Thus, having determined the causal inference of the air pollutants (Table 8), it could be inferred that not every correlation implies causation; however, every causation implies correlation. Therefore, starting with a correlation analysis involves risks in the case of the proposed model. Thus, another explanation could be as a result of random chance, as the variables may appear to be related, but have no true underlying and clear relationships.

The LSTM-GAM-xAI model produced some detailed statistical information such as the coefficient, standard error, *z*-score, *p*-value and confidence interval. Again, number of observations, residual, Pearson Chi-square, etc. (Table 4). The post-hoc explanation model, such as LIME, produced information on features within their threshold, predicted values within a min and max range, and feature values. In view of this, the GAM provides more interpretation than the LIME model. Kaur et al. (2020) indicates that GAM is more interpretable than post-hoc explanation models like SHAP (Kaur et al., 2020). Thus, our study also goes further to suggest that GAM provides more statistical information compared to LIME. Therefore, by proposing and experimenting with the hybrid LSTM-GAM-xAI model for pollutant prediction, detailed information is provided for human understanding. Thus, irrespective of the M-day time steps, our model adapted to the dataset and provided further information on air pollutant analysis for Kimberley.

Though the causal inference model was applied with methodological rigor, the model’s resulting effect estimates (Table 9) produced high *p*-values (e.g., >0.8) indicating no statistically significant relationships, therefore any conclusion drawn must be interpreted with caution.

While the performance metrics such as MSE, RMSE and MAE provide useful insight, it also emphasizes the baseline to evaluate the technical accuracy of the proposed model in decision making. The interpretability of the output and feasibility of integrating the model in real-world scenarios are essential considerations in assessing whether the model can inform timely interventions in the real world. Although the proposed model achieved relatively low MSE value generally, as compared to the other models, it however shows promising performance results in different time-steps.

Our research contributes in the context of air pollution prediction a transparent and interpretable model that stakeholders could trust. It is worth noting, that researchers have provided several models in different context. For example, Zhu et al. (2024) provided spatio-temporal model based on machine learning for prediction of NO; Lin et al. (2025) incorporated LSTM with GAM and Bayesian model for prediction of different air pollutants; while Goudet et al. (2018) focused on cause-effect inference with the causal generative neural network. The advantage of our model over the existing models is that, ours provide interpretability through the integration of emerging explainable AI model such as LIME with existing GAM and LSTM, thereby bridging the gap between traditional statistical model and deep learning model such as LSTM in a predictive task during environmental monitoring of air pollutants. Furthermore, our model may serve as the alternative to legacy-based air pollutants prediction systems available at air quality monitoring stations in the Northern Cape Province of the Republic of South Africa.

Our proposed model improves interpretability compared to other deep learning models such as LSTM, because it also considered existing statistical models such as GAM, which provides information on coefficient (*coef*), standard error (*std err*), *z*-score, *p*-value and confidence interval. This information is used by statisticians to understand the behavior of models used in modeling environmental data. Typical LIME or SHAP models lack this information which our model now provides. Therefore, our model provides added value by combining the GAM with post-hoc explanation in terms of transparency and actionable insight for practitioners in environmental monitoring.

## 6 Methodological limitation

The use of synthetized data based on the measurements from Hantam station for the model evaluation was one of the limitations of this study. As this was due to the lack of air pollutants data for Kimberley. Thus, our research bridges this gap to help evaluate the proposed model’s behavior in a data-scarce context. Again, though using data from one source (e.g., Hantam) to another location (that is, Kimberley) could introduce a potential bias due to site-specific meteorological and geographical differences, this concern was addressed by evaluating the models performance against multiple air pollutants.

## 7 Conclusion

Air pollutants emission is a global issue as they contribute to global warming and climate-related activities. Human activities like burning fossil fuels contribute to air pollution and climate change. In this study, a deep learning time-series model was proposed that leverages an LSTM model with GAM and offers post-hoc explanation with causal inference analysis of common air pollutants such as PM_2.5_, PM_10_, O_3_, SO_2_, NO_2_, NO, NO_x_. Meteorological factors were also considered as input into the proposed model, in addition to these air pollutants. Random sampling method was adopted to synthesis dataset for the City of Kimberley. The local interpretable model-agnostic explanations, which is an artificial intelligence technique, provides a local interpretation of individual predictions. Different time-step previous data, such as 5-day and 10-day, were considered, and MSE, MAE, RMSE values were used to evaluate the predictions. The results indicate better performance value when compared with comparative models. Thus, the study is beneficial to air monitoring stations as it attempts to present alternate models for air pollutant concentration prediction. By conducting this research, we provide an effective approach to assist with the hybridization of the state-of-the-art deep learning time-series model, which could be used as an alternative to a legacy-based air quality prediction model for the city of Kimberley in the Province of Northern Cape, in the Republic of South Africa. Future research should consider the proposed model as one of the emerging models in air pollutant concentration prediction to further fine-tune the predictions in existing real world models.

## Data Availability

The raw data supporting the conclusions of this article will be made available by the authors, without undue reservation.

## Glossary

xAI: explainable artificial intelligence
LIME: local interpretable model-agnostic explanation
NOx: nitrogen oxides
NO_2_: nitrogen dioxide
NO: nitric oxide
CO: carbon monoxide
O_3_: ozone
PM_10_ & PM_2.5_: particulate matter
SO_2_: sulfur dioxide
LSTM: long short-term memory model
GAM: generalized additive model
SHAP: Shapley additive explanations
1DCNN: one-dimensional convolutional neural network
BiLSTM: Bi-directional long short-term memory
GRU: gated recurrent unit
ARIMA: autoregressive integrated moving average
CNN: convolutional neural network
LightGBM: light gradient boosting machine
SAAQIS: South African Air Quality Information System
NAQI: Northern Cape National Air Quality Indicator

## References

[ref1] Adamkiewicz Ł. Maciejewska K. Rabczenko D. Drzeniecka-Osiadacz A. (2022). Ambient particulate air pollution and daily hospital admissions in 31 cities in Poland. Atmos. 13:345. doi: 10.3390/atmos13020345

[ref2] Agbehadji I. E. Obagbuwa I. C. (2024). Systematic review of machine learning and deep learning techniques for spatiotemporal air quality prediction. Atmosphere, MDPI 15:1352. doi: 10.3390/atmos15111352

[ref3] Agbehadji I. E. Obagbuwa I. C. (2025a). Mode decomposition bi-directional long short-term memory (BiLSTM) attention mechanism and transformer (AMT) model for ozone (O3) prediction in Johannesburg, South Africa. Forecasting 7, 1–19. doi: 10.3390/forecast7020015

[ref4] Agbehadji I. E. Obagbuwa I. C. (2025b). Integration of explainable artificial intelligence into hybrid long short-term memory and adaptive Kalman filter for sulfur dioxide (SO2) prediction in Kimberley, South Africa. Atmos. 16:523. doi: 10.3390/atmos16050523 PMC12358413 40832676

[ref5] Bai Y. Ni Y. Zeng Q. (2021). Impact of ambient air quality standards revision on the exposure-response of air pollution in Tianjin, China. Environ. Res. 198:111269. doi: 10.1016/j.envres.2021.111269, PMID: 33945811

[ref6] Barbalat G. Hough I. Dorman M. Lepeule J. Kloog I. (2024). A multi-resolution ensemble model of three decision-tree-based algorithms to predict daily NO2 concentration in France 2005–2022. Environ. Res. 257:119241. doi: 10.1016/j.envres.2024.119241, PMID: 38810827

[ref7] Becker D. Alfeus A. Molnár P. Boman J. Wichmann J. (2024). Ambient PM2.5, soot, black carbon and organic carbon levels in Kimberley, South Africa. Clean Air J 34:100. doi: 10.17159/caj/2024/34/2.20100

[ref8] Bitz T. Gerling L. Meier F. Weber S. (2024). Prediction of urban ultrafine particle emission fluxes using generalized additive models. Atmos. Environ. 334:120677. doi: 10.1016/j.atmosenv.2024.120677, PMID: 40689378

[ref9] Chang C.-H. Caruana R. Goldenberg A. (2022). NODE-GAM: neural generalized additive model for interpretable deep learning. USA: Cornell University.

[ref10] Charumbira S. Ncube A. (2022). An environmental disaster: a critical review of kimberlite diamond Mining in Kimberley, South Africa. Int J Disaster Risk Reduct:20. doi: 10.2139/ssrn.4232987

[ref11] Cheng M. Fang F. Navon I. M. Pain C. (2023). Ensemble Kalman filter for GAN-ConvLSTM based long lead-time forecasting. J Comput Sci 69:102024. doi: 10.1016/j.jocs.2023.102024, PMID: 40689378

[ref12] Cheng B. Ma Y. Feng F. Zhang Y. Shen J. Wang H.. (2021). Influence of weather and air pollution on concentration change of PM2.5 using a generalized additive model and gradient boosting machine. Atmos. Environ. 255:118437. doi: 10.1016/j.atmosenv.2021.118437, PMID: 40689378

[ref13] Dammann L. M. Freitag M. Thielmann A. Säfken B. (2025). Gradient-based smoothing parameter estimation for neural P-splines. Comput. Stat. 40, 3645–3663. doi: 10.1007/s00180-024-01593-z

[ref14] de Asís López F. Ordóñez C. Roca-Pardiñas J. (2024). A generalized additive model (GAM) approach to principal component analysis of geographic data. Spatial Statistics 59:100806. doi: 10.1016/j.spasta.2023.100806

[ref15] de Foy B. Edwards R. Joy K. S. Zaman S. U. Salam A. Schauer J. J. (2024). Interpretable machine learning tools to analyze PM2.5 sensor network data so as to quantify local source impacts and long-range transport. Atmos. Res. 311:107656. doi: 10.1016/j.atmosres.2024.107656, PMID: 40689378

[ref16] Department of Environmental Affairs. (2019). National air quality indicator - monthly data report for the northern Cape Province. p. 1. South Africa. Available at: https://saaqis.environment.gov.za/Pagesfiles/Northern%20Cape%20-%20February%202019.pdf

[ref17] Dillon E. LaRiviere J. Lundberg S. Roth J. Syrgkanis V. Be careful when interpreting predictive models in search of causal insights. A joint article about causality and interpretable machine learning with 2018. (2025). Available online at: https://shap.readthedocs.io/en/latest/example_notebooks/overviews/Be%20careful%20when%20interpreting%20predictive%20models%20in%20search%20of%20causal%20insights.html. (Accessed 20 March, 2025).

[ref18] Eslami E. Choi Y. Lops Y. Sayeed A. (2020). A real-time hourly ozone prediction system using deep convolutional neural network. Neural Comput. & Applic. 32, 8783–8797. doi: 10.1007/s00521-019-04282-x

[ref19] Fan X. Jie X. Zou F. Wang D. da H. Li H.. (2024). Association between outdoor air pollutants and risk of acute exacerbation of chronic obstructive pulmonary disease in Xi’an, China. Air Qual. Atmos. Health 17, 1373–1390. doi: 10.1007/s11869-024-01513-6

[ref20] Fang C. Zhou Z. Li J. Zhou M. Chen X. (2021). Short-term nitrogen dioxide exposure is associated with the spread of *S. pyogenes*-induced vulvovaginitis in prepubertal girls in Hangzhou, China. Environ. Sci. Pollut. Res. 28, 35790–35797. doi: 10.1007/s11356-021-13268-z, PMID: 33677663

[ref21] Forestry Fisheries and the Environment (2024). National air Quality Indicator - monthly data report for the northern Cape Province. South Africa: Department Forestry, Fisheries and the Environment, Republic of South Africa, 1–15.

[ref22] Fu J. Liu Y. Zhao Y. Tang S. Chen Y. Liu Y.. (2023). Hourly Valley concentration of air pollutants associated with increased acute myocardial infarction hospital admissions in Beijing, China. Atmos. 14:27. doi: 10.3390/atmos14010027

[ref23] Fu G. An T. Liu H. Tian Y. Wang P. (2020). Assessment of the impact of PM2.5 exposure on the daily mortality of circulatory system in Shijiazhuang, China. Atmos. 11:1018. doi: 10.3390/atmos11091018

[ref24] Gao A. You X. Li Z. Liao C. Yin Z. Zhang B.. (2025). Health effects associated with ozone in China: a systematic review. Environ. Pollut. 367:125642. doi: 10.1016/j.envpol.2025.125642, PMID: 39761714

[ref25] Goudet O. Kalainathan D. Caillou P. Guyon I. Lopez-Paz D. Sebag M. (2018). Learning functional causal models with generative neural networks. Xplainable and interpretable models in computer vision and machine learning, 39–80.

[ref26] Gu J. Shi Y. Chen N. Wang H. Chen T. (2020). Ambient fine particulate matter and hospital admissions for ischemic and hemorrhagic strokes and transient ischemic attack in 248 Chinese cities. Sci. Total Environ. 715:136896. doi: 10.1016/j.scitotenv.2020.136896, PMID: 32007884

[ref27] Habeebullah T. M. A. (2020). Assessment of ground-level ozone pollution with monitoring and modelling approaches in Makkah, Saudi Arabia. Arab. J. Geosci. 13:1164. doi: 10.1007/s12517-020-06179-9

[ref28] Haddad K. Vizakos N. (2021). Air quality pollutants and their relationship with meteorological variables in four suburbs of greater Sydney, Australia. Air Qual. Atmos. Health 14, 55–67. doi: 10.1007/s11869-020-00913-8

[ref29] Hammouda Z. Zaier L. H. Blond N. (2021). Modeling tropospheric ozone and particulate matter in Tunis, Tunisia using generalized additive model. Clean Air J 31, 1–16. doi: 10.17159/caj/2021/31/2.8880

[ref30] He M. Z. Yitshak-Sade M. Just A. C. Gutiérrez-Avila I. Dorman M. de Hoogh K.. (2023). Predicting fine-scale daily NO2 over Mexico city using an ensemble modeling approach. Atmospheric. Pollut. Res. 14:101763. doi: 10.1016/j.apr.2023.101763, PMID: 37193345 PMC10168642

[ref31] Huang C. S. Lo K. Wu Y. L. Wang F. C. Shiu Y. S. Chen C. C.. (2025). Estimating and characterizing spatiotemporal distributions of elemental PM2.5 using an ensemble machine learning approach in Taiwan. Atmospheric. Pollut. Res. 16:102463. doi: 10.1016/j.apr.2025.102463, PMID: 40689378

[ref32] Hwang J.-S. Gil J.-W. Lee C.-K. (2024). Determination of optimal batch size of deep learning models with time series data. Sustainability 16:5936. doi: 10.3390/su16145936

[ref33] Jo W. Kim D. (2023). Neural additive time-series models: explainable deep learning for multivariate time-series prediction. Expert Syst. Appl. 228:120307. doi: 10.1016/j.eswa.2023.120307, PMID: 40689378

[ref34] Kaur H. Nori H. Jenkins S. Caruana R. Wallach H. Vaughan Wortman J.. (2020). “Interpreting interpretability: understanding data scientists' use of interpretability tools for machine learning” in Proceedings of the 2020 CHI conference on human factors in computing systems (Honolulu HI USA: ACM), 1–14.

[ref35] Kruschel S. Hambauer N. Weinzierl S. Zilker S. Kraus M. Zschech P. (2025). Challenging the performance-interpretability trade-off: an evaluation of interpretable machine learning models. Bus. Inf. Syst. Eng., 1–25. doi: 10.1007/s12599-024-00922-2, PMID: 40689350

[ref36] LeCun Y. Bengio Y. Hinton G. (2015). Deep learning. Nature 521, 436–444. doi: 10.1038/nature14539, PMID: 26017442

[ref37] Lin Y. C. Lin Y. T. Chen C. R. Lai C. Y. (2025). Meteorological and traffic effects on air pollutants using Bayesian networks and deep learning. J. Environ. Sci. (China) 152, 54–70. doi: 10.1016/j.jes.2024.01.057, PMID: 39617575

[ref38] Lou Y. Caruana R. Gehrke J. (2012). “Intelligible models for classification and regression” in Proceedings of the 18th ACM SIGKDD international conference on knowledge discovery and data mining (Beijing: ACM Press), 150.

[ref39] Naz F. Fahim M. Cheema A. A. Viet N. T. Cao T. V. Hunter R.. (2024). Two-stage feature engineering to predict air pollutants in urban areas. IEEE Access 12, 114073–114085. doi: 10.1109/ACCESS.2024.3443810

[ref40] Nisbet R. Elder J. Miner G. (2009). “Chapter 7 basic algorithms for data mining: a brief overview” in Handbook of statistical analysis and data mining applications, 121–150.

[ref41] Obster F. Brand J. Ciolacu M. Humpe A. (2022). Improving boosted generalized additive models with random forests: a zoo visitor case study for smart tourism. Procedia Comput. Sci. 217, 187–197. doi: 10.1016/j.procs.2022.12.214, PMID: 40690924

[ref42] Ortega-Fernandez I. Sestelo M. Villanueva N. M. (2024). Explainable generalized additive neural networks with independent neural network training. Stat. Comput. 34:6. doi: 10.1007/s11222-023-10320-5, PMID: 40689350

[ref43] Ravindra K. Rattan P. Mor S. Aggarwal A. N. (2019). Generalized additive models: building evidence of air pollution, climate change and human health. Environ. Int. 132:104987. doi: 10.1016/j.envint.2019.104987, PMID: 31398655

[ref44] Setianingrum A. H. Anggraini N. Ikram M. F. D. (2022). “Prophet model performance analysis for Jakarta air quality forecasting” in 2022 10th international conference on cyber and IT service management (CITSM). doi: 10.1109/CITSM56380.2022.9936037

[ref45] Sharma G. Khurana S. Saina N. Shivansh Gupta G. (2024). Comparative analysis of machine learning techniques in air quality index (AQI) prediction in smart cities. Int. J. Syst. Assur. Eng. Manag. 15, 3060–3075. doi: 10.1007/s13198-024-02315-w, PMID: 40689350

[ref46] Srivastava N. Hinton G. Krizhevsky A. Sutskever I. Salakhutdinov R. (2014). Dropout: a simple way to prevent neural networks from overfitting. J Mach Learn Res 15, 1929–1958. doi: 10.5555/2627435.2670313

[ref47] Talati I. Shanmugam R. Shah K. Chaudhary N. Parikh K. Khaitan D. (2023). “Air pollution monitoring & prediction system for Ahmedabad region using IOT and ML” in 2023 14th International Conference on Computing Communication and Networking Technologies (ICCCNT).

[ref48] Taormina R. Mesin L. Orione F. Pasero E. (2011). “Forecasting tropospheric ozone concentrations with adaptive neural networks” in The 2011 International Joint Conference on Neural Networks.

[ref49] Tejada-Lapuerta A. Bertin P. Bauer S. Aliee H. Bengio Y. Theis F. J. (2025). Causal machine learning for single-cell genomics. Nat. Genet. 57, 797–808. doi: 10.1038/s41588-025-02124-2, PMID: 40164735

[ref50] Tian R. Li X. Ma Z. Liu Y. Wang J. Wang C. (2023). LDformer: a parallel neural network model for long-term power forecasting. Front Inform Technol Electron Eng. 24, 1287–1301. doi: 10.1631/FITEE.2200540

[ref51] Tu Q. Hase F. Chen Z. Schneider M. García O. Khosrawi F.. (2023). Estimation of NO2 emission strengths over Riyadh and Madrid from space from a combination of wind-assigned anomalies and a machine learning technique. Atmos. Meas. Tech. 16, 2237–2262. doi: 10.5194/amt-16-2237-2023, PMID: 38859159

[ref52] Tyralis H. Papacharalampous G. (2024). A review of predictive uncertainty estimation with machine learning. Artif. Intell. Rev. 57:94. doi: 10.1007/s10462-023-10698-8

[ref53] Wahiduzzaman M. Yeasmin A. (2024). A generalised additive model and deep learning method for cross-validating the North Atlantic oscillation index. Atmos. 15:987. doi: 10.3390/atmos15080987

[ref54] Wattal K. Singh S. K. (2021). “Multivariate air pollution levels forecasting” in 2021 2nd International Conference on Advances in Computing, Communication, Embedded and Secure Systems (ACCESS). doi: 10.1109/ACCESS51619.2021.9563281

[ref55] Widiputra H. Mailangkay A. Gautama E. (2021). Multivariate CNN-LSTM model for multiple parallel financial time-series prediction, vol. 54. doi: 10.1155/2021/9903518Citations

[ref56] Xiong K. Xie X. Huang L. Hu J. (2024). Improved O3 predictions in China by combining chemical transport model and multi-source data with machine learning techniques. Atmos. Environ. 318:120269. doi: 10.1016/j.atmosenv.2023.120269, PMID: 40689378

[ref57] Zang Z. Guo Y. Jiang Y. Zuo C. Li D. Shi W.. (2021). Tree-based ensemble deep learning model for spatiotemporal surface ozone (O3) prediction and interpretation. Int J Appl Earth Observat Geoinform 103:102516. doi: 10.1016/j.jag.2021.102516, PMID: 40689378

[ref58] Zhang A. Yang J. Wang F. (2023). Application and enabling digital twin technologies in the operation and maintenance stage of the AEC industry: a literature review. J Build Eng 80:107859. doi: 10.1016/j.jobe.2023.107859

[ref59] Zhu Q. Lee D. Stoner O. (2024). A comparison of statistical and machine learning models for spatio-temporal prediction of ambient air pollutant concentrations in Scotland. Environ. Ecol. Stat. 31, 1085–1108. doi: 10.1007/s10651-024-00635-5

