# Supplementary material for: A hybrid long short-term memory with generalized additive model and post-hoc explainable artificial intelligence with causal inference for air pollutants prediction in Kimberley, South Africa
Source: Front Artif Intell. 2025 Aug 4;8:1620019. doi: 10.3389/frai.2025.1620019 (PMC12358413; doi:10.3389/frai.2025.1620019)
Supplement: Supplementary file 1 [file Data_Sheet_1.docx]

# **Appendix A1: LSTM-GAM-xAI statistical information on air pollutants for 5-day time step.**

| **Dependent variable** | **Independent Variable** | **Coef** | **Std err** | **z** | **p>\|z\|** | **[0.025** | **0.975]** |
| --- | --- | --- | --- | --- | --- | --- | --- |
| PM_10 Prediction_ | **Const** | 1.8041 | 0.271 | 6.649 | 0.000 | 1.272 | 2.336 |
|  | NO_2_ | -0.0186 | 0.034 | -0.540 | 0.589 | -0.086 | 0.049 |
|  | NO | 0.0174 | 0.031 | 0.551 | 0.581 | -0.044 | 0.079 |
|  | NOx | -0.0246 | 0.028 | -0.878 | 0.380 | -0.080 | 0.030 |
|  | CO | 0.0091 | 0.206 | 0.044 | 0.965 | -0.395 | 0.413 |
|  | SO_2_ | 0.0015 | 0.043 | 0.036 | 0.971 | -0.083 | 0.086 |
|  | PM_2.5_ | 0.0059 | 0.033 | 0.177 | 0.859 | -0.059 | 0.071 |
|  | O_3_ | -0.005 | 0.002 | -0.242 | 0.809 | -0.004 | 0.003 |
|  | WS | 0.0042 | 0.003 | 1.618 | 0.106 | -0.001 | 0.009 |
|  | AT | 0.0011 | 0.006 | -0.175 | 0.861 | -0.013 | 0.011 |
|  | RH | -0.0014 | 0.002 | -0.731 | 0.465 | -0.005 | 0.002 |
|  | SR | 2.7x10^-0.5^ | 0.000 | 0.185 | 0.853 | -0.000 | 0.000 |
|  |  |  |  |  |  |  |  |
| PM_2.5_ prediction | Const | 1.0789 | 0.235 | 4.592 | 0.000 | 0.618 | 1.539 |
|  | NO_2_ | -0.0243 | 0.030 | -0.824 | 0.410 | -0.082 | 0.034 |
|  | NO | -0.0317 | 0.027 | -1.173 | 0.241 | -0.085 | 0.021 |
|  | NOx | -0.0369 | 0.024 | 1.534 | 0.125 | -0.010 | 0.084 |
|  | CO | 0.3265 | 0.176 | -1.850 | 0.064 | -0.672 | 0.019 |
|  | SO_2_ | 0.0232 | 0.037 | 0.625 | 0.532 | -0.050 | 0.096 |
|  | PM_10_ | 0.0043 | 0.025 | 0.177 | 0.859 | -0.044 | 0.052 |
|  | O_3_ | -0.0017 | 0.002 | -1.006 | 0.314 | -0.005 | 0.002 |
|  | WS | 0.0016 | 0.002 | 0.721 | 0.471 | -0.003 | 0.006 |
|  | AT | 0.0096 | 0.005 | 1.854 | 0.064 | -0.001 | 0.020 |
|  | RH | 0.0013 | 0.002 | 0.824 | 0.410 | -0.002 | 0.004 |
|  | SR | -2.39x10^-05^ | 0.000 | -0.189 | 0.0850 | -0.000 | 0.000 |
|  |  |  |  |  |  |  |  |
| NOx prediction | Const | 4.4534 | 0.251 | 17.749 | 0.000 | 3.962 | 4.945 |
|  | NO_2_ | -0.0277 | 0.035 | -0.791 | 0.429 | -0.096 | 0.041 |
|  | NO | -0.0675 | 0.032 | 2.109 | 0.034 | 0.005 | 0.130 |
|  | NOx | -0.0520 | 0.034 | 1.534 | 0.125 | -0.014 | 0.118 |
|  | CO | -0.1033 | 0.210 | -0.493 | 0.622 | -0.514 | 0.308 |
|  | SO_2_ | -0.0316 | 0.044 | 0.718 | 0.473 | -0.055 | 0.118 |
|  | PM_10_ | -0.0255 | 0.029 | -0.878 | 0.380 | -0.083 | 0.031 |
|  | O_3_ | 2.967x10^-06^ | 0.002 | 0.001 | 0.999 | -0.004 | 0.004 |
|  | WS | -0.0002 | 0.003 | 0.069 | 0.945 | -0.005 | 0.005 |
|  | AT | -0.0002 | 0.006 | -0.035 | 0.972 | -0.012 | 0.012 |
|  | RH | -0.0017 | 0.002 | 0.886 | 0.375 | -0.002 | 0.005 |
|  | SR | 4.986x10^-05^ | 0.000 | 0.332 | 0.740 | -0.000 | 0.000 |
|  |  |  |  |  |  |  |  |
| NO_2_ prediction | Const | 3.8854 | 0.201 | 19.371 | 0.000 | 3.492 | 4.278 |
|  | NO | -0.0002 | 0.026 | -0.009 | 0.993 | -0.051 | 0.051 |
|  | NOx | -0.0184 | 0.023 | -0.791 | 0.429 | -0.064 | 0.027 |
|  | PM_10_ | -0.0228 | 0.028 | -0.824 | 0.410 | -0.077 | 0.031 |
|  | CO | -0.1528 | 0.171 | 0.894 | 0.371 | -0.182 | 0.488 |
|  | SO_2_ | -0.0268 | 0.036 | 0.748 | 0.455 | -0.044 | 0.097 |
|  | PM_10_ | -0.0128 | 0.024 | -0.540 | 0.589 | -0.059 | 0.034 |
|  | O_3_ | -0.0015 | 0.002 | 0.900 | 0.368 | -0.002 | 0.005 |
|  | WS | -0.0044 | 0.002 | 2.055 | 0.040 | 0.000 | 0.009 |
|  | AT | -0.0037 | 0.005 | 0.738 | 0.461 | -0.006 | 0.014 |
|  | RH | -0.0011 | 0.002 | 0.743 | 0.458 | -0.002 | 0.004 |
|  | SR | 5.592x10^-05^ | 0.000 | 0.457 | 0.648 | -0.000 | 0.000 |
|  |  |  |  |  |  |  |  |
| CO prediction | Const | 0.1175 | 0.038 | 3.078 | 0.002 | 0.043 | 0.192 |
|  | NO | 0.0039 | 0.004 | 0.890 | 0.373 | -0.005 | 0.012 |
|  | NO_2_ | 0.0043 | 0.005 | 0.894 | 0.371 | -0.005 | 0.014 |
|  | NOx | -0.0019 | 0.004 | -0.493 | 0.622 | -0.010 | 0.006 |
|  | PM_2.5_ | -0.0085 | 0.005 | -1.850 | 0.064 | -0.018 | 0.001 |
|  | SO_2_ | -0.0041 | 0.006 | -0.680 | 0.496 | -0.016 | 0.008 |
|  | PM_10_ | 0.0002 | 0.004 | 0.044 | 0.965 | -0.008 | 0.008 |
|  | O_3_ | 0.0002 | 0.000 | 0.905 | 0.366 | -0.000 | 0.001 |
|  | WS | -0.0007 | 0.000 | -1.915 | 0.056 | -0.001 | 1.63e-05 |
|  | AT | -0.0003 | 0.001 | -0.309 | 0.757 | -0.002 | 0.001 |
|  | RH | 6.903x 10^-05^ | 0.000 | 0.267 | 0.789 | -0.000 | 0.001 |
|  | SR | -6.447 x10^-06^ | 2.05 x10^-05^ | -0.315 | -0.753 | 4.66 x10^-05^ | 3.37 x10^-05^ |

# **Appendix A2: LSTM-GAM-xAI statistical information of air pollutant for 10 day time-step.**

| **Dependent variable** | **Independent Variable** | **Coef** | **Std err** | **z** | **p>\|z\|** | **[0.025** | **0.975]** |
| --- | --- | --- | --- | --- | --- | --- | --- |
| NO_2 Prediction_ | **Const** | 3.8809 | 0.203 | 19.165 | 0.000 | 3.484 | 4.278 |
|  | NO | -2.294x10^-05^ | 0.026 | -0.001 | 0.999 | -0.052 | 0.051 |
|  | NOx | -0.0176 | 0.024 | -0.742 | 0.458 | -0.064 | 0.029 |
|  | PM_2.5_ | -0.0236 | 0.028 | -0.850 | 0.395 | -0.078 | 0.031 |
|  | CO | -0.1508 | 0.172 | 0.879 | 0.379 | -0.185 | 0.487 |
|  | SO_2_ | -0.0271 | 0.036 | 0.754 | 0.451 | -0.043 | 0.098 |
|  | PM_10_ | -0.0124 | 0.024 | -0.521 | 0.602 | -0.059 | 0.034 |
|  | O_3_ | -0.0014 | 0.002 | 0.881 | 0.378 | -0.002 | 0.005 |
|  | WS | -0.0045 | 0.002 | 2.070 | 0.038 | 0.000 | 0.009 |
|  | AT | -0.0037 | 0.005 | 0.731 | 0.465 | -0.006 | 0.014 |
|  | RH | -0.0012 | 0.002 | 0.759 | 0.448 | -0.002 | 0.004 |
|  | SR | 5.538x10^-05^ | 0.000 | 0.451 | 0.652 | -0.000 | 0.000 |
|  |  |  |  |  |  |  |  |
| NO prediction | Const | 1.2187 | 0.249 | 4.888 | 0.000 | 0.730 | 1.707 |
|  | NO_2_ | -2.725x10^-05^ | 0.031 | -0.001 | 0.999 | -0.061 | 0.061 |
|  | NOx | 0.0603 | 0.026 | 2.335 | 0.020 | 0.010 | 0.111 |
|  | PM_2.5_ | -0.0314 | 0.030 | -1.039 | 0.299 | -0.091 | 0.028 |
|  | CO | 0.1827 | 0.187 | 0.977 | 0.328 | -0.184 | 0.549 |
|  | SO_2_ | 0.0008 | 0.039 | 0.020 | 0.984 | -0.076 | 0.078 |
|  | PM_10_ | 0.0117 | 0.026 | 0.452 | 0.652 | -0.039 | 0.063 |
|  | O_3_ | 0.0010 | 0.002 | 0.565 | 0.572 | -0.002 | 0.005 |
|  | WS | 0.0004 | 0.002 | 0.169 | 0.866 | -0.004 | 0.005 |
|  | AT | 0.0046 | 0.006 | 0.835 | 0.404 | -0.006 | 0.015 |
|  | RH | 0.0014 | 0.002 | 0.834 | 0.404 | -0.002 | 0.005 |
|  | SR | -0.0001 | 0.000 | -0.975 | 0.330 | -0.000 | 0.000 |
|  |  |  |  |  |  |  |  |
| NOx prediction | Const | 4.4794 | 0.247 | 18.113 | 0.000 | 3.995 | 4.964 |
|  | NO_2_ | -0.0256 | 0.035 | -0.742 | 0.458 | -0.093 | 0.042 |
|  | NO | -0.0738 | 0.032 | 2.335 | 0.020 | 0.012 | 0.136 |
|  | PM_2.5_ | -0.0434 | 0.033 | 1.296 | 0.195 | -0.022 | 0.109 |
|  | CO | -0.0773 | 0.207 | -0.374 | 0.709 | -0.483 | 0.328 |
|  | SO_2_ | -0.0340 | 0.043 | 0.783 | 0.434 | -0.051 | 0.119 |
|  | PM_10_ | -0.0257 | 0.029 | -0.895 | 0.371 | -0.082 | 0.031 |
|  | O_3_ | -0.0002 | 0.002 | 0.083 | 0.934 | -0.004 | 0.004 |
|  | WS | -0.0004 | 0.003 | -0.142 | 0.887 | -0.005 | 0.005 |
|  | AT | -0.0014 | 0.006 | -0.229 | 0.819 | -0.013 | 0.011 |
|  | RH | -0.0013 | 0.002 | 0.676 | 0.499 | -0.002 | 0.005 |
|  | SR | 7.716x10^-05^ | 0.000 | 0.521 | 0.603 | -0.000 | 0.000 |
|  |  |  |  |  |  |  |  |
| PM_2.5_ prediction |  |  |  |  |  |  |  |
|  | NO_2_ | 0.0755 | 0.041 | 1.832 | 0.067 | -0.005 | 0.156 |
|  | NO | -78.4156 | 90.587 | -0.866 | 0.387 | -255.963 | 99.132 |
|  | NOx | -0.0004 | 0.025 | -0.015 | 0.988 | -0.050 | 0.049 |
|  | CO | -0.0657 | 0.049 | -1.339 | 0.181 | -0.162 | 0.030 |
|  | SO_2_ | -0.0419 | 0.028 | -1.522 | 0.128 | -0.096 | 0.012 |
|  | PM_10_ | -0.0074 | 0.028 | -0.262 | 0.793 | -0.062 | 0.048 |
|  | O_3_ | 0.0278 | 0.025 | 1.103 | 0.270 | -0.022 | 0.077 |
|  | WS | 0.0059 | 0.024 | 0.246 | 0.806 | -0.041 | 0.053 |
|  | AT | 0.0454 | 0.024 | 1.863 | 0.062 | -0.002 | 0.093 |
|  | RH | 0.0076 | 0.024 | 0.310 | 0.757 | -0.040 | 0.055 |
|  | SR | 0.0317 | 0.024 | 1.306 | 0.191 | -0.016 | 0.079 |
|  |  |  |  |  |  |  |  |
| PM_10_ prediction |  |  |  |  |  |  |  |
|  | NO_2_ | -0.0262 | 0.044 | -0.591 | 0.555 | -0.113 | 0.061 |
|  | NO | -33.9832 | 92.524 | -0.367 | 0.713 | -215.327 | 147.361 |
|  | NOx | 0.0167 | 0.026 | 0.639 | 0.523 | -0.034 | 0.068 |
|  | CO | 0.0137 | 0.050 | 0.274 | 0.784 | -0.084 | 0.112 |
|  | SO_2_ | -0.0223 | 0.027 | -0.812 | 0.417 | -0.076 | 0.032 |
|  | PM_2.5_ | 0.0087 | 0.030 | 0.293 | 0.769 | -0.049 | 0.067 |
|  | O_3_ | -0.0008 | 0.025 | -0.032 | 0.974 | -0.049 | 0.048 |
|  | WS | -0.0162 | 0.025 | -0.658 | 0.511 | -0.064 | 0.032 |
|  | AT | 0.0110 | 0.024 | 0.455 | 0.649 | -0.037 | 0.059 |
|  | RH | 0.0416 | 0.025 | 1.646 | 0.100 | -0.008 | 0.091 |
|  | SR | 0.0060 | 0.025 | 0.239 | 0.811 | -0.043 | 0.055 |
|  |  |  |  |  |  |  |  |
| O_3_ prediction |  |  |  |  |  |  |  |
|  | NO_2_ | -0.0234 | 0.047 | -0.500 | 0.617 | -0.115 | 0.068 |
|  | NO | 35.3817 | 102.619 | 0.345 | 0.730 | -165.748 | 236.511 |
|  | NOx | 0.0057 | 0.028 | 0.202 | 0.840 | -0.050 | 0061 |
|  | CO | -0.0955 | 0.056 | -1.719 | 0.086 | -0.204 | 0.013 |
|  | SO_2_ | 0.0298 | 0.031 | 0.954 | 0.340 | -0.031 | 0.091 |
|  | PM_2.5_ | 0.0357 | 0.032 | 1.103 | 0.270 | -0.028 | 0.099 |
|  | PM_10_ | -0.0682 | 0.032 | -2.146 | 0.032 | -0.130 | -0.006 |
|  | WS | -0.0170 | 0.027 | -0.621 | 0.535 | -0.071 | 0.037 |
|  | AT | -0.0123 | 0.028 | -0.443 | 0.657 | -0.066 | 0.042 |
|  | RH | 0.0434 | 0.028 | 1.572 | 0.116 | -0.011 | 0.098 |
|  | SR | 0.0354 | 0.028 | 1.287 | 0.198 | -0.019 | 0.089 |
|  |  |  |  |  |  |  |  |
|  |  |  |  |  |  |  |  |
| CO prediction | Const | 0.1124 | 0.038 | 2.925 | 0.003 | 0.037 | 0.188 |
|  | NO | 0.0043 | 0.004 | 0.977 | 0.328 | -0.004 | 0.013 |
|  | NO2 | 0.0042 | 0.005 | 0.879 | 0.379 | -0.005 | 0.014 |
|  | NOx | -0.0015 | 0.004 | -0.374 | 0.709 | -0.009 | 0.006 |
|  | PM_2.5_ | -0.0086 | 0.005 | -1.860 | 0.063 | -0.018 | 0.000 |
|  | SO2 | -0.0042 | 0.006 | -0.699 | 0.485 | -0.016 | 0.008 |
|  | PM_10_ | 0.0005 | 0.004 | 0.119 | 0.905 | -0.007 | 0.008 |
|  | O3 | 0.0003 | 0.000 | 0.962 | 0.336 | -0.000 | 0.001 |
|  | WS | -0.0007 | 0.000 | -1.896 | 0.058 | -0.001 | 2.29x10^-05^ |
|  | AT | -0.0002 | 0.001 | -0.247 | 0.805 | -0.002 | 0.001 |
|  | RH | 7.823x10^-05^ | 0.000 | 0.302 | 0.762 | -0.000 | 0.001 |
|  | SR | -5.065x10^-06^ | 2.05x10^-05^ | -0.247 | 0.805 | -4.53x10^-05^ | 3.51x10^-05^ |

# **Appendix A3: LSTM-GAM-xAI display of LIME information on air pollutants with 5-day time-step.**

| 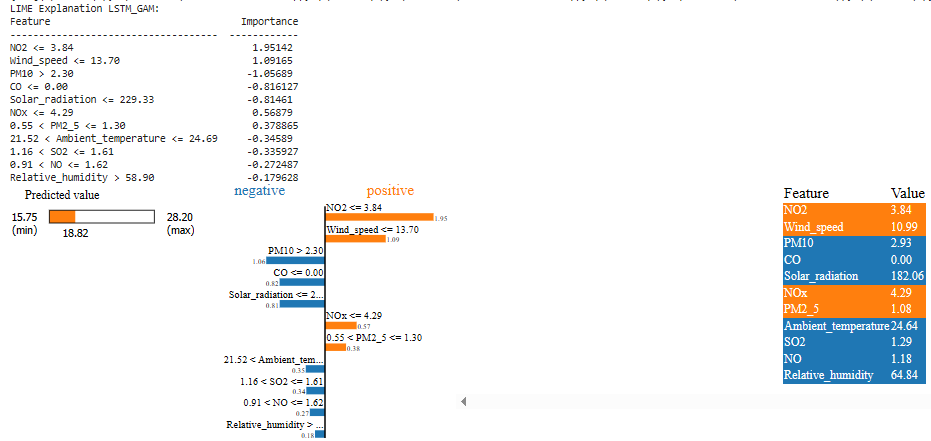 |
| --- |
| 1. O_3_ prediction |
| 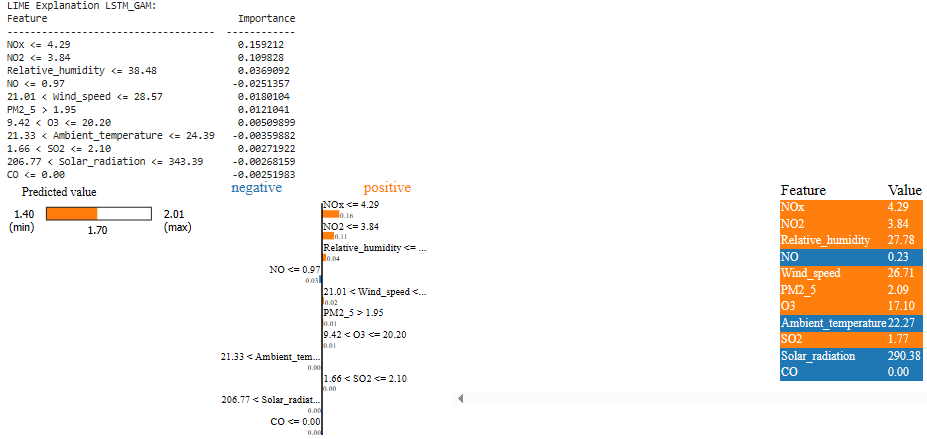 |
| 1. PM_10_ prediction |
| 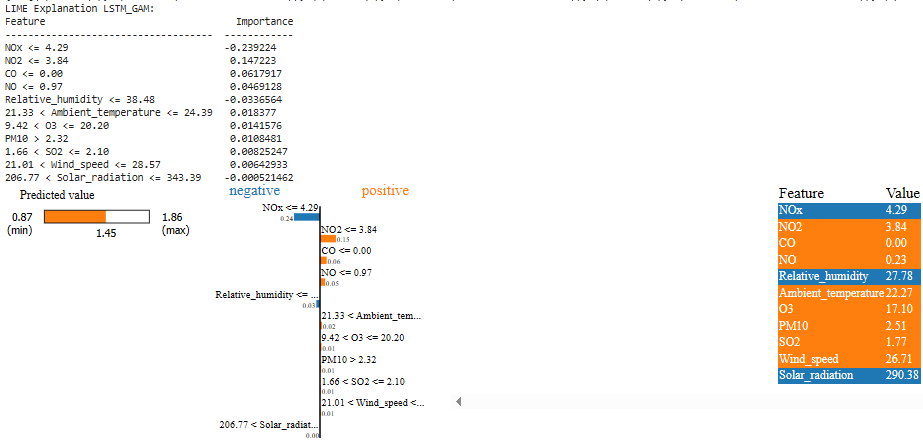 |
| 1. PM_2.5_ prediction |
| 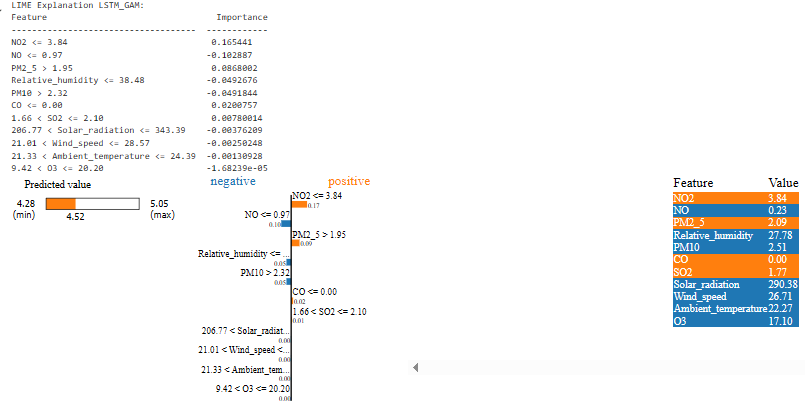 |
| 1. NOx prediction |
| 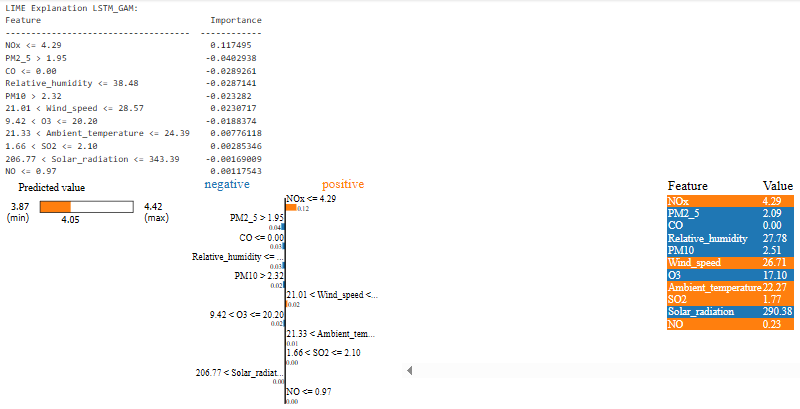 |
| 1. NO_2_ prediction |


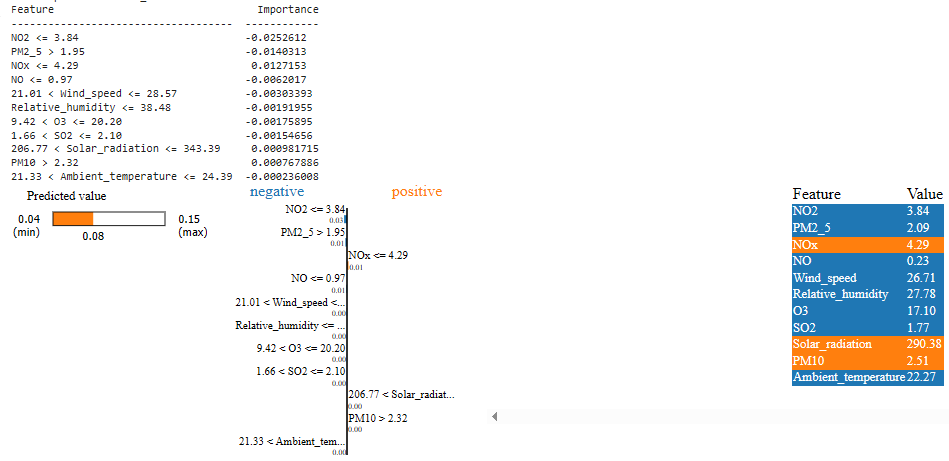


1. CO prediction

# **Appendix A4: LSTM-GAM-xAI display of LIME information on air pollutants with 10-day time-step.**

| 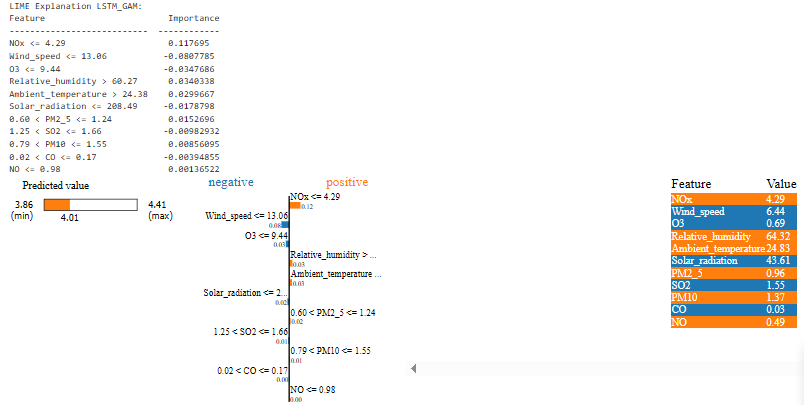 |
| --- |
| 1. NO_2_ prediction |
| 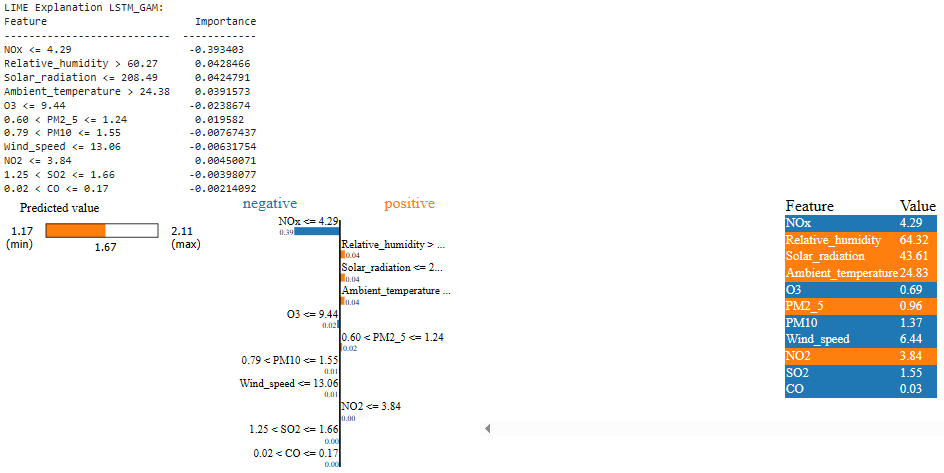 |
| 1. NO prediction |
| 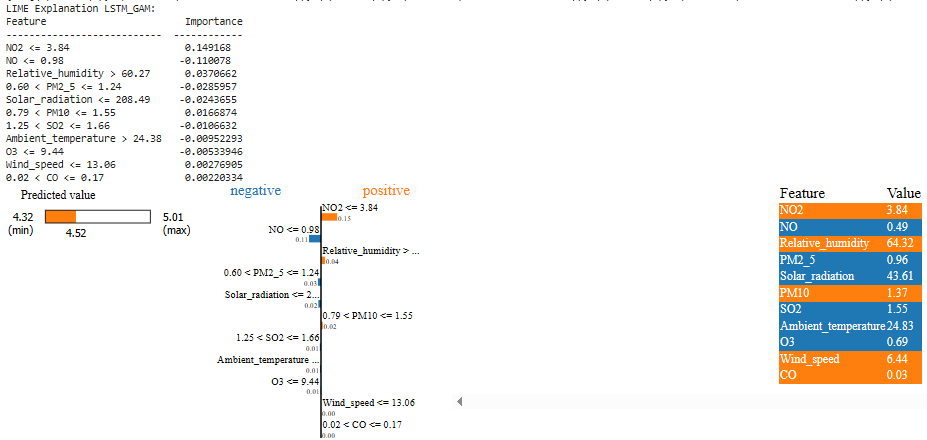 |
| 1. NOx prediction |
| 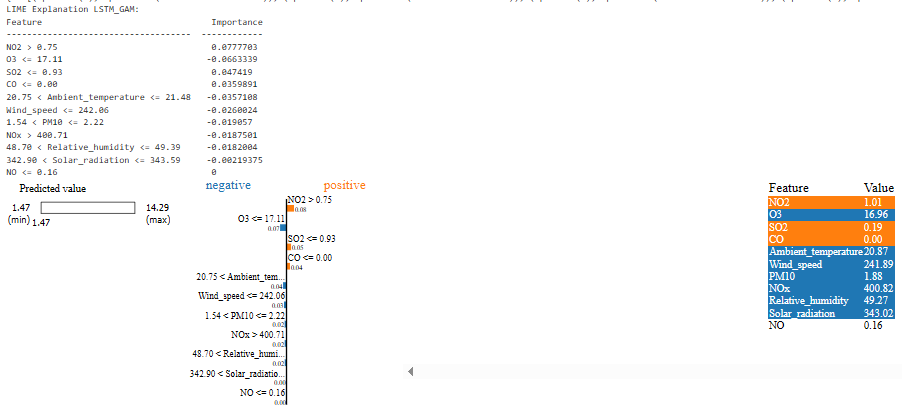 |
| 1. PM_2.5_ prediction |
| 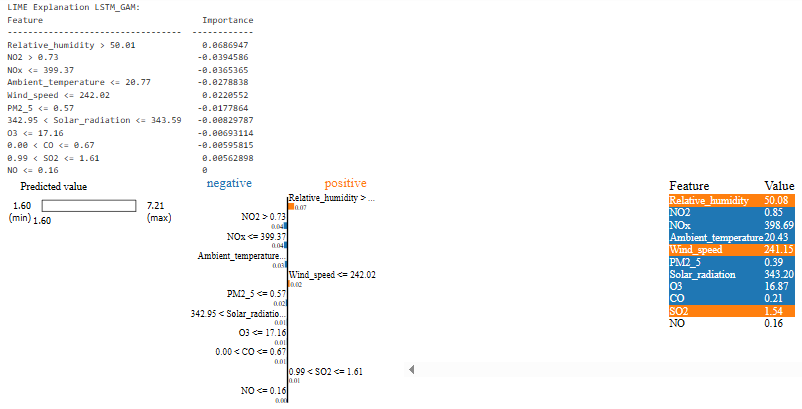 |
| 1. PM_10_ prediction |
| 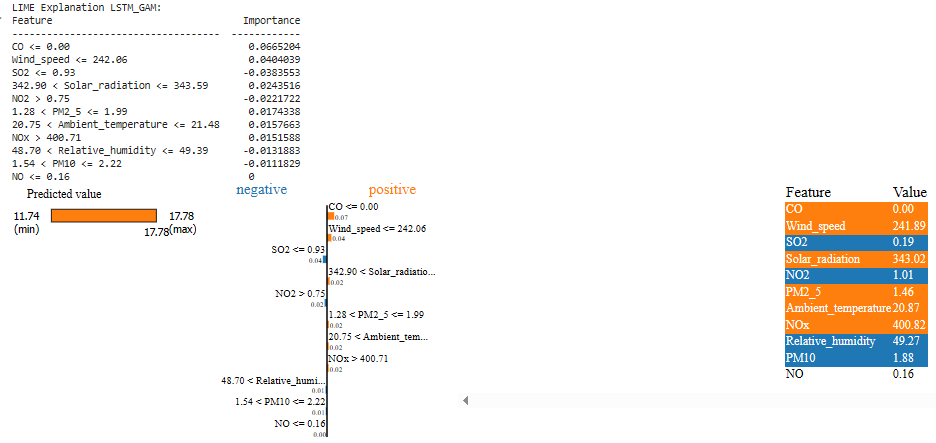 |
| 1. O_3_ prediction |
